# Data Mining by Pluralistic Approach on CRISPR Gene Editing in Plants

**DOI:** 10.3389/fpls.2019.00801

**Published:** 2019-07-09

**Authors:** Tanushri Kaul, Nitya Meenakshi Raman, Murugesh Eswaran, Arulprakash Thangaraj, Rachana Verma, Sonia Khan Sony, Krishnamurthy M. Sathelly, Rashmi Kaul, Pranjal Yadava, Pawan Kumar Agrawal

**Affiliations:** ^1^Nutritional Improvement of Crops Group, International Centre for Genetic Engineering and Biotechnology, New Delhi, India; ^2^Department of Biotechnology, Indian Institute of Maize Research, Indian Institute of Agricultural Biotechnology (ICAR), New Delhi, India; ^3^National Agricultural Science Fund, Indian Council of Agricultural Research, New Delhi, India

**Keywords:** genome, sgRNA, double-stranded break, non-homologous end joining repair, homology-directed repair, Cas9, Cas13, C2c2

## Abstract

Genome engineering by site-specific nucleases enables reverse genetics and targeted editing of genomes in an efficacious manner. Contemporary revolutionized progress in targeted-genome engineering technologies based on Clustered Regularly Interspaced Short Palindromic Repeats (CRISPR)-related RNA-guided endonucleases facilitate coherent interrogation of crop genome function. Evolved as an innate component of the adaptive immune response in bacterial and archaeal systems, CRISPR/Cas system is now identified as a versatile molecular tool that ensures specific and targeted genome modification in plants. Applications of this genome redaction tool-kit include somatic genome editing, rectification of genetic disorders or gene therapy, treatment of infectious diseases, generation of animal models, and crop improvement. We review the utilization of these synthetic nucleases as precision, targeted-genome editing platforms with the inherent potential to accentuate basic science “strengths and shortcomings” of gene function, complement plant breeding techniques for crop improvement, and charter a knowledge base for effective use of editing technology for ever-increasing agricultural demands. Furthermore, the emerging importance of Cpf1, Cas9 nickase, C2c2, as well as other innovative candidates that may prove more effective in driving novel applications in crops are also discussed. The mined data has been prepared as a library and opened for public use at www.lipre.org.

## Introduction

The Clustered Regularly Interspaced Short Palindromic Repeats (CRISPR) system is largely involved in conferring resistance to genetic transformants, and viruses, if present in surrounding environment, rendering a type of acquired immunity. The system was first recognized in *Escherichia coli* in 1987, but the relativity of its biological function was only completely understood 18 years later when its role was confirmed in rendering adaptive immunity (Ishino et al., 1987; Bolotin et al., 2005; Mojica et al., 2005; Pourcel et al., 2005). Within the next 2 years, CRISPR favoring antiviral protection was also confirmed (Barrangou et al., 2007). In the years thereafter, the combination of CRISPR with CRISPR associated (Cas) genes was demonstrated for RNA mediated DNA targeting in the immune system (Brouns et al., 2008; Marraffini and Sontheimer, 2008; Garneau et al., 2010; Deltcheva et al., 2011).

Clustered Regularly Interspaced Short Palindromic Repeats system analyzed in *Streptococcus pyogenes* consists of three genes – Cas9 nuclease, non-coding RNA genes viz. the pre CRISPR targeting RNA (pre-crRNA) and the *trans-*activating crRNA (tracr-RNA) (Jinek et al., 2012; Xing et al., 2014; Weeks et al., 2016). A mechanism involving the action of CRISPR/Cas defense comprises of three stages, adaptation, immunization, and spacer acquisition (Vander et al., 2009; Garneau et al., 2010; Horvath and Barrangou, 2010; Karginov and Hannon, 2010; Marraffini and Sontheimer, 2010; Bhavya et al., 2011). This adaptive immunity of the bacterial system is now making previously well-established and fully well-developed technologies outdates as it is relatively quick, less expensive and less cumbersome.

## Mechanisms of Gene Editing Using CRISPR

Traditional gene editing is particularly challenging and relies on the mechanism of homologous recombination. The low frequency of spontaneous recombination associated makes traditional approaches intrinsically inefficient and labor intensive as it requires the specific use of antibiotic selection and other techniques to identify the rare cells in which the mutagenesis is successful. Advanced genome editing facilitates the knock-out of a gene or knock-in a specific variant by introducing double stranded breaks (DSBs) at a desired site of the genome that dramatically increases the efficiency of mutagenesis. This dramatic increase in efficacy is exponentially utilized by research groups dealing with plants, fruit flies, mammalian cells, and invertebrates.

There are two methods that largely are involved in repairing DSBs – non-homologus end joining (NHEJ) and homology directed repair (HDR). The former deals with a simple innate mechanism of rejoining the two free ends without any specificity and is therefore error-prone and often results in the Indel mutations at the repair sites. The other method, HDR, utilizes the sister chromatid/chromosome as the repair template to cleanly replace the area of break via homologous recombination. HDR is therefore, well-preferred as it can be used to introduce a DNA vector of interest as a repair template. Alternatively, even ssDNA oligonucleotides that matches the sequence around the DSB can be used as repair template. The DNA vector or ssDNA oligonucleotides can carry the mutation of interest in the middle of their sequence and site-specific mutagenesis can be achieved.

**FIGURE 1:**
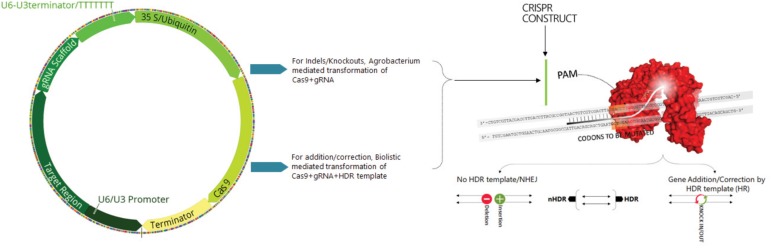
Scheme illustrating the construction of Cas9+sgRNA cassette, sgRNA structure and mechanism of the target recognition. Cas9 endonuclease is guided to the target DNA called protospacer (orange) by sgRNA contains a 20 nt spacer (red). PAM sequence (green) is essential for binding and chopping. The two domains of Cas9 RuvC and HNH each cut one strand of a double stranded DNA (brown). Targeted genome editing in eukaryotic species mediated by site specific nucleases (SSNs). The induced double strand break by SSNs can be repaired by either error prone NHEJ (rejoins the broken ends of DNA with random insertions or deletions) or HDR (providing a donor DNA sequence resulting in gene addition or correction).

The basic mechanism of the CRISPR system involves the incorporation of specific small fragments of foreign or non-self-nucleic acids between short DNA repeats in the host genome which, in conjunction with the Cas proteins, recognize the incoming foreign nucleic acids and destroy them. Briefly, mature crRNA is formed in combination with tracr-RNA processing pre-crRNA containing identical directs containing spacers. The two non-coding RNA genes can be substituted by gRNA that incorporated a designed hairpin useful in mimicking the cr-RNA-track-RNA complex. The specificity of Cas9 with the target DNA is determined by both a protospacer adjacent motif (PAM) sequence that lies immediately downstream of the target region and a gRNA-DNA base pairing (Figure 1; Xing et al., 2014). Cas9 proteins specifically generate DSBs by stimulating the cellular repair process and increase the efficiency of HDR.

**FIGURE 2:**
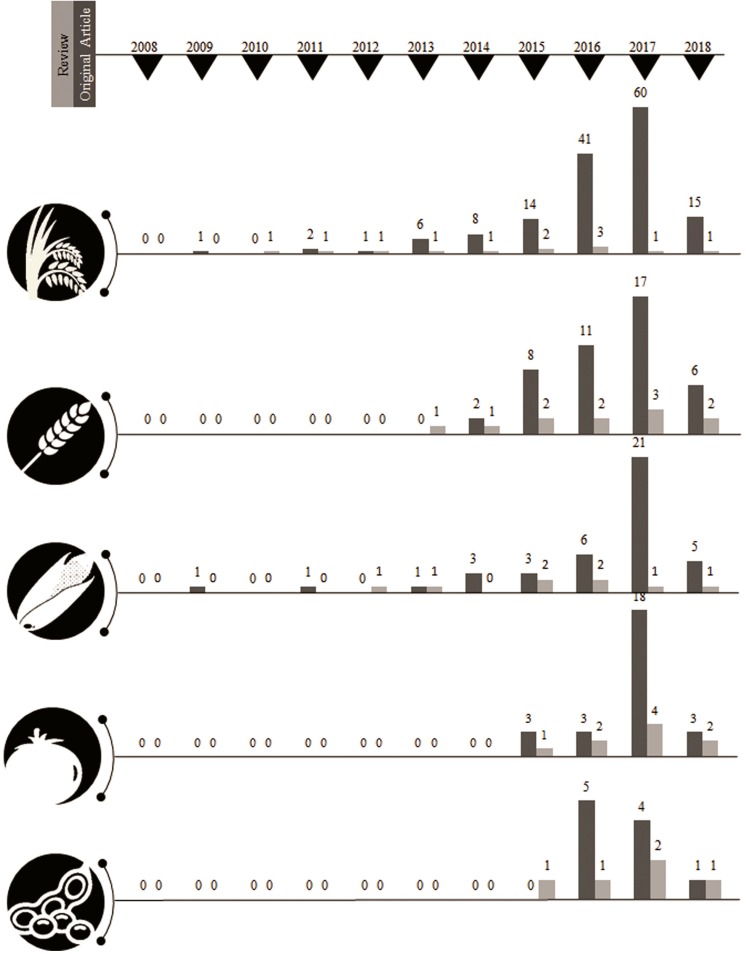
Outliers signify, steady divergence in research progress influenced by review articles published up to the same year. Articles have been collected based on training keywords for pseudorandomness. Accuracy prediction is about >0.2 threshold of total NCBI retrieve. The list of total articles retrieved from the trained dataset is attached in the Supplementary Materials with its corresponding hyperlink.

Although the efficiency of CRISPR/Cas9 system heavily relies on Cas9 proteins serving as an RNA guide system, it is the CRISPR locus that functions as a genetic memory. The locus primarily constitutes a single-guide chimeric RNA (sg-RNA) of the CRISPR/Cas9 system, created by fusing cr-RNA with track-RNA (Voytas, 2013). The uniqueness of Cas proteins involved in the CRISPR/Cas system is evidenced by the classification made by Makarova et al. (2011b) (Figure 2). Based on specific Cas proteins, CRISPR/Cas systems were categorized into types I, II, and III (Table 1). Despite the presence of Cas1 and Cas2 proteins in all the three types of CRISPR/Cas system (Makarova et al., 2011a), the variation among different CRISPR/Cas systems is bought about by the effector complex that triggers cleavage by binding to crRNA. Other differences associated among the three CRISPR/Cas systems such as their origin, types of components and nature of target is briefly discussed in Table 1. Targeting of DNA sequences by the type I system is carried out with the assistance of the endonuclease activity of the Cas3 protein (Makarova et al., 2011a,b). The type II CRISPR/Cas system that includes Cas1, Cas2, Cas9, and Cas4/Csn2 types have been reported only in bacteria as an elementary system consisting of four proteins. The type III CRISPR/Cas system is recognized by the Cas1, Cas2, Cas10, and Cas6 proteins.

**TABLE 1:** Different classes of the CRISPR/Cas systems and their unique features.

**Type**	**Organism**	**Component/Protein**	**Cleavage domain of proteins**	**Target**
Type I	*Escherichia coli*, *Pseudomonas aeruginosa*	Cas1, Cas2, Cas3, Cas5, Cas6, Cas7	HD nuclease domain of Cas3	DNA
Type II	*Streptococcus thermophilus*	Cas1, Cas2, Cas9, Cas4/Csn2	RuvC-like nuclease domain near the N terminus and HNH (McrA-like) nuclease domain in the middle of Cas9	DNA
Type III	*Staphylococcus epidermis*, *Lactococcus lactis*, and *Pyrococcus furiosus*	Cas1, Cas2, Cas10, Cas6	Catalytic triad of Cas6 protein and Csm/Cmr Complex	DNA/RNA

Genome targeted studies in Archaea, yeast, bacteria, animals, plants, and human cell lines have been proven to be achieved by CRISPR system (Xing et al., 2014). As CRISPR has been an efficient and simple RNA-guided endonuclease technology, subsequent germ-line transmission due to gene mutations have been achieved (Li D. et al., 2013; Wang et al., 2013; Xing et al., 2014). *In vitro* effortlessness in translating CRISPR/Cas9 technology for plant-based research has been observed with a steady rise due to its easy to construct design and assembly.

## The Transition From ZFNs and TALENs to CRISPR

Zinc finger nucleases (ZFNs) and transcription activator-like effector nucleases (TALENs) have been used successfully so far for simultaneous gene-based editing of multiple genes in many crops (Supplementary Material S1). Techniques like ZFNs and TALENs largely promoted functional genomic studies to address fundamental development related queries in plants, plant growth and their response to the environment. ZFNs consist of multiple zinc finger domain bearing proteins generated from the common Cys-2-His 2-zinc finger domain (Porteus and Carroll, 2005) capable of recognizing a specific sequence (Weeks et al., 2016). The composition of each Zinc finger motif binds to three nucleotides and is made up of almost 30 amino acids. The gene editing efficiency of ZFNs was first reported successful in *Arabidopsis* species (Lloyd et al., 2005).

TALENs have derived from the transcription activator-like effectors (TALEs) produced by plant pathogen *Xanthomonas* species. Prior to the development of facile TALENs methods in plants including demonstration of TALENs-stimulated homologous recombination leading to gene replacement (Cermak et al., 2011; Li et al., 2012; Reyon et al., 2012; Christian et al., 2013; Zhang et al., 2013; Mahfouz et al., 2014; Weeks et al., 2016), successful gene editing by TALENs was first demonstrated in yeast (Li et al., 2011; Christian et al., 2013). A year later effective heritable mutations were generated in tomatoes (Lor et al., 2014). CRISPR/Cas9 system has more advantages over ZFNs and TALENs because of minimal unintended modifications and cellular toxicity, efficiency, target design simplicity. Due to several advantages, ZFNs and TALENs are being replaced by the CRISPR/Cas9 system as it consists of a single monomeric protein and a chimeric RNA. The versatility of the CRISPR system heavily lies on Cas9 protein, which operates to independently bind and DNA sequence-dependent cleavage (Anders et al., 2014; Jinek et al., 2014; Nishimasu et al., 2014; Sternberg et al., 2014). CRISPR systems are promptly superseding ZFNs/TALENs, owing to their ease of use and the hold of the promise to advance crop improvement, and address problems related to increased agricultural demands (Woo et al., 2015). CRISPR/Cas9 systems only require an NGG PAM motif to be present downstream of the target sequence and the gRNA should be chosen carefully to avoid cleavage of off targets. With the assistance to *in silico* modules, prediction of PAM regions is made easier by analyzing the genome sequences of *Arabidopsis*, soybean, *Medicago truncatula*, tomato, rice, maize, *Brachypodium distachyon*, and *sorghum* with higher specificity of predicted gRNAs among monocots (Xie et al., 2014).

## gRNAs and Pam Capitalization for CRISPR/Cas9 System

In the simplified mechanism of CRISPR that is now being utilized in plant cells, the RNA component is guide-RNA (gRNA), which can be up to 100 nucleotides in length. The Cas protein component has a nuclease activity that remains the same regardless of target DNA and binds to the gRNA which hybridizes to a ssDNA component. Cas9 also binds to several adjacent nucleotides in the genome; thus, a triple complex of DNA, RNA and protein is involved. The specificity of the complex is encoded in the first 20 nucleotides of the gRNA. Simply put, by changing the 20-nucleotide sequence one can change the DNA sequence to which the gRNA can bind. Once bound, a DSB is induced in the DNA component of the complex. The duration of less than a day to make a new guide RNA in the laboratory and the multiplexing of the Cas9 with more than one gRNA matching two or three gene sequences facilitates simultaneous targeting of multiple sites in the genome.

The adaptation of the CRISPR/Cas9 system to plants is rendered by the use of different PAM requirements with Cas9 homologs. The limitation of using the standard full-length gRNAs can be circumvented by the choice of targets without the requirement of either Adenine or Guanidine at the 5′ end making the transcripts produced to be one nucleotide longer in length (Hwang et al., 2013; Cho et al., 2014). In this regard, an artificial gene containing ribozyme sequences were transcribed from any promoter that resulted in the production of gRNAs which self-catalyzed the cleavage of the primary transcript (Gao and Zhao, 2014). Similar experimentation was also adapted by Upadhyay et al. (2013) and Jia and Wang (2014) in plants to eliminate 5′ end transcript-based restriction. This adaptation of production of the gRNAs results in the possibility of not limiting well characterized inducible promoters, developmental stage or tissue-specific differential expression of multiple gRNAs. Several gRNAs targeting the same gene in a human cell line showed that average content of GC was related to efficient targeting when compared to gRNAs with high or low GC content (Malina et al., 2015). Therefore, we now have started working on the development of a web tool to design gRNAs for effective targeting of both monocot and dicot genes. Although the mutation rate of the CRISPR/Cas9 system depends on the sensitivity of the analytical method (restriction analysis and/or sequencing), cell type and delivery method, the influences of gRNAs sequence/structure and gRNA expression strategy can be made clear by using our database.

## Diversity in the CRISPR System

CRISPR/Cpf1 (CRISPR from *Prevotella* and *Francisella*) is a novel genome-editing tool, which makes staggered cuts, resulting in a 5′ overhang that improves the frequency of DNA insertions. Cpf1 cleaves at a distal site, which preserves the seed region that is essential for target recognition. T-rich protospacer-adjustment motif makes Cpf1 better suited to editing A-T-rich DNA than Cas9, which has a G-rich protospacer adjustment motif. Cpf1 is easy to deliver to cells as it is a smaller homolog of Cas9 and does not require a tra-crRNA (Attar, 2015). Cpf1 contains a RuVC nuclease domain, which is segmented into three components (Sontheimer and Wolfe, 2015). It holds the potential to be used for a wide variety of experiments where shorter RNA species are useful, for example, editing A-T rich genomes, and its application can range from therapeutic treatment to agricultural products. Also, recently Cpf1 proved its specificity by producing a targeted mutant mouse (Kim and Kim, 2014). In 2015, Zetsche and coworkers established Cpf1 was more advantageous than Cas9. First, Cpf1 generates cleavage products with staggered cuts as opposed to blunt end cutting by the Cas9. The staggered end cutting results in an improved precision of DNA insertions due to the 5′ overhangs. Secondly, the seed region essential for target recognition and future editing is established by Cpf1 influenced cutting at the distal end. Thirdly, the T-rich PAM makes Cpf1 better suited to editing than G-rich PAM of Cas9. Therefore, it can be believed that better efficiency can be achieved by CRISPR with its companion Cpf1 (Zetsche et al., 2015).

Cas13 (formerly called C2c2) proteins that possess two enzymatically distinct RNase activities are classified as Cas13a, Cas13b, Cas13c, and Cas13d. Recently, 2 years of research have revealed that Cas13 cleaves the direct repeat of CRISPR-RNA (crRNA) in a pre-crRNA array to form a complex of Cas13-crRNA. There is a possibility of Cas13 being sold as an RNA guided RNA targeting effector as it contains higher eukaryotes and prokaryotes nucleotide-binding (HEPN) domains which are generally RNases (Anantharaman et al., 2013; Shmakov et al., 2015). It is believed to target RNA because *in vitro* analysis of C2c2 revealed it was guided by a single crRNA to achieve interference (Abudayyeh et al., 2016). In contrast to other RNAs, which feature the presence of two HEPN domains aids C2c2 in cleaving RNA (Benda et al., 2014). Applications of C2c2 varies from visualization of localisation and trafficking of RNA capture specific transcripts for destruction (Abudayyeh et al., 2016), by providing a mechanism for RNA detection and impactful diagnostic applications (Gootenberg et al., 2017, 2018). For effective application of C2c2 in biology, it is imperative to comprehend its programmable RNA binding and cleavage activity. Reports of specific RNA cleavage in plant cell lines established by Aman et al. (2018) underscored the understanding of the mechanism involved in the specific binding of Cas13. A rational design of crRNAs with optimal specificity and activity by Tambe et al. (2018) emphasizes the consideration of Cas13 off-target recognition.

A mutated version of Cas9 called as Cas9n (nickase) is responsible for single-strand break (SSB), nicking DNA at a specific location instead of cleaving it as in the case of Cas9, which gets repaired in a cell by homology directed repair (HDR) (Riordan et al., 2015). When these nicks are made at adjacent sites on two opposite strands it causes DSB, creating a 5′ or 3′ overhangs along the target. Mutagenesis of catalytic residues in Cas9 (D10A in RuvC and H840A in HNH) was used to produce Cas9n to reduce potential off-targets (Jinek et al., 2012; Nishimasu et al., 2014). This technology has been utilized effectively for producing desired effects with increased specificity in rice (Ran et al., 2013).

Cytosine deaminase and adenosine deaminase mediated CRISPR/Cas9 base editing technology is capable of efficiently and precisely introducing point mutations without any donor templates or dsDNA breaks and this can be applied in diverse genera of plants, human, yeasts, and mammals (Komor et al., 2016; Nishida et al., 2016; Lu and Zhu, 2017; Ren et al., 2018). Such targeted base editing in crops without the need of foreign DNA donor or dsDNA cleavage was discussed by Shimatani et al. (2017). Targeted conversion of C to T in protoplasts and plants of rice, wheat and maize was validated by Zong et al. (2017) in and in watermelon (Tian et al., 2018) using CRISPR/Cas9 nickase- cytidine deaminase toolkit (Zong et al., 2017). G-C and A-T conversions were validated in protoplasts of regenerated rice and wheat by utilizing (Li et al., 2018a), the efficiency of cytosine and adenosine base editors to enable single-nucleotide conversions in a reversible manner without dsDNA cleavage has been recognized as a new dimension in genome editing (Kim, 2018).

Among plants, the main strategy employed to increase the editing efficiency of the CRISPR/Cas9 system is the use of strong promoters and to avoid usage of a low-scored guide sequence (Hu et al., 2018). Although the robust and widely used *S. pyogenes* Cas9 (SpCas9) requires sites containing NGG PAMs, the expansion in the range of CRISPR/Cas9 genome editing has involved the screening of many variants or orthologs of Cas9 proteins such as *Streptococcus thermophilus* CRISPR1/Cas9 targeting NNAGAAW PAMs (Deveau et al., 2008), *S. thermophilus* CRISPR3/Cas9 for NGGNG PAMs (Horvath et al., 2008), *Neisseria meningitides* Cas9 for NNNNGATT PAMs (Zhang et al., 2013), VQR and VRER variants of SpCas9 targeting NGA and NGCG PAMs, respectively (Kleinstiver et al., 2015). Recent studies are therefore, targeted to increase the efficiency of these variants on par with the wild type Cas9 by expressing the variants with strong endogenous promoters. A similar approach was conducted in rice by using ACT1 and UBI1 promoters and the observations resulted in a ∼fourfold increase of mutation rate using UBI1 promotor whereas ACT1 increases the mutation rate by an average of approximately sixfold (Hu et al., 2018).

## Anomaly Detection in CRISPR Technology

### Timeline Infographics of CRISPR Specific Publication Seed

A year-wise timeline for the frequency of publications for the past decade revealed a phenomenal augmentation in the interest of the research community with a CRISPR system for genome editing among plants. The sparse generation of data using CRISPR system until the year 2012 could be attributed to standardizing the experimentation of genome editing with respect to various factors such as biotic, abiotic, and enzyme related effects. More than a single fold increase was observed between the years 2013 and 2017 sequentially with the present year of 2018 already generating 315 publications among food and fodder related crops. The CRISPR system has been estimated in a record of 87 plants until 2017 revealing that the adaptation of this genome editing technology has been achieved with minimum off-target mutations by many research groups worldwide. The detailing of the CRISPR-based genome editing was well discussed in many review articles and specifically in a total of nine articles for tomato, seven for rice, six for wheat, five for maize, and two articles for soybean until March 2018 (Supplementary Material S2). Original research articles that maintain the pulse of research increments in the field of genome editing revealed CRISPR system to be extensively investigated in rice (60 publications in the year 2017 alone) followed by wheat, tomato, maize and soybean (Table 2).

**TABLE 2:** Trained keyword parsing from NCBI PubMed.

**2008**	**2009**	**2010**	**2011**	**2012**	**2013**	**2014**	**2015**	**2016**	**2017**	**2018**	**CRISPR specific to**	**Type of retrieval**	**Consolidated seed**
0	0	0	0	0	0	0	0	5	4	1	Soybean		10
0	1	0	1	0	1	3	3	6	21	5	Wheat		41
0	0	0	0	0	0	0	3	3	18	3	Tomato	Original article	27
0	1	0	2	1	6	8	14	41	60	15	Rice		148
0	0	0	0	0	0	2	8	11	17	6	Maize		44
0	0	0	0	0	0	0	1	1	2	1	Soybean		5
0	0	0	0	1	1	0	2	2	1	1	Wheat		8
0	0	0	0	0	0	0	1	2	4	2	Tomato	Review	9
0	0	1	1	1	1	1	2	3	1	1	Rice		12
0	0	0	0	0	1	1	2	2	3	2	Maize		11
1	2	1	3	2	5	30	44	87	112	28	Crop	Crop specific	315
1	2	1	2	6	15	51	151	252	383	133	Plants	Total	982

*Timeline plotted against each year from 2008 based on the influence of review articles to do basic and advance research by using CRISPR genome editing tools.*

### Vector Collections Used in Plant Expression System Using CRISPR/Cas9

The exciting application of CRISPR/Cas9 assay is the experimentation with a pool of vectors that are capable to inhibit or invoke ∼10 k gRNA sequences that can be cloned using libraries of up to 200 base pairs (Gilbert et al., 2013; Shalem et al., 2014; Sanjana et al., 2014). Introduction of a single viral sequence as per a single CRISPR construct per cell is feasible for protein-coding gene-based studies but falls short for non-protein coding elements as paired gRNAs are required in this regard. For non-protein coding gene vector systems (Jinek et al., 2012) that express two gRNAs compatible with oligonucleotide library and obtained from a single plasmid (Derrien et al., 2012) cloning would be a fulfilling approach. gRNAs introduced into the host cells by a plasmid vector either via viral infection or transfection generally encompasses the scaffold within the expression plasmid. Some deletion experiments have been conducted by co-transfecting independent plasmids with two separate gRNAs by expressing a single gRNA (Chen et al., 2014; Zheng et al., 2014; Ho et al., 2015). A recent protocol has been devised by Aparicio-Prat et al. (2015) for simultaneous cloning of two distinct gRNAs into a single lentiviral vector. The two vector CRISPR/Cas9 system allows for faster knockout of the target gene in cells by first selecting cells expressing high levels of Cas9, and is also great for creating custom gRNA libraries for screening assays. Enhancement of CRISPR efficiency focused on the usage of efficient gRNAs, designing of novel binary vectors. This work by Durr et al. (2018) demonstrates high efficiency can be engineered by combining these strategies with direct plant regeneration along with intergenic regulatory sequences, and heritable targeted chromosomal deletions of large gene clusters. An overview of the plasmids involved in CRISPR experimentation is mentioned (Table 3 and Supplementary Material S3).

**TABLE 3:** Summary of CRISPR-plasmids used for transcriptional activation and generation of sequence-specific gRNAs.

**Name**	**Published by/in**
pRGEB32	Xie et al., 2015
pRGE32	
pYPQ131D2.0	Lowder et al., 2018
pYPQ141A2.0	
pYPQ132C2.0	
pYPQ132B2.0	
pYPQ132D2.0	
pYPQ141B2.0	
pYPQ141D2.0	
pYPQ132A2.0	
pYPQ133B2.0	
pYPQ133D2.0	
pYPQ141C2.0	
pYPQ131A2.0	
pYPQ133A2.0	
pYPQ133C2.0	
pHdzCas9-KRAB	Kao and Ng,2017
pTX179	Tang et al., 2016
pTX168	
pTX172	
pBAtC	Kim et al., 2016
pHAtC	
pHDE-35S-Cas9-mCherry	Gao et al., 2016
pHDE-35S-Cas9-mCherry-UBQ	
pJG85	Gil-Humanes et al., 2017
pSC6	Curtin et al., 2018
pSC12	
pSC5	
pTX171	Tang et al., 2016
pTX176	

### Temperature Correspondence and Modulations of CRISPR System

Engineering targeted mutations using the CRISPR/Cas9 system has been universally accepted. However, efficiency of targeted mutations largely depends on standardizing the factors that will negate off-target mutations. The study by LeBlanc et al. (2018) reported higher frequencies of on-target mutations when *Arabidopsis* sp. were subjected to heat stress at 37°C when compared to quantitative GFP based experimentation at 22°C. An inter-genus comparison between the *Citrus* and *Arabidopsis* plants by the same authors in the same publication revealed the temperature dependent efficiency of on-target mutations by CRISPR/Cas9 system at 37°C.

Abiotic stresses such as high temperature affects photosynthetic machinery, thereby, affecting the yield of food crops. Qiu et al. (2018) revealed interesting observations by integrating CRISPR/Cas9 system in comparing the localization and greening phenotype between the wild-type and *hsa1* mutant phenotypes of rice. *hsa1* mutant induced by CRISPR/Cas9 system, in this study, was heat sensitive with reduced expression of plastid genes but had a faster greening phenotype.

Stable homozygous mutants generated by CRISPR/Cas9 system in lettuce was analyzed in primary and secondary transformants by targeting *LsNCED4* (9-*cis-*epoxycarotenoid dioxygenase 4) responsible for influencing temperature-based inhibition in seed germination. This study by Bertier et al. (2018) revealed increases in seed germination efficiency among primary and secondary homozygous cultivars to the tune of more than 70% at 37°C concluding that enrichment of gene-based editing in germlines could be simply achieved by germinating the seeds of the mutated phenotypes at high temperature.

Modulation of related events can be achieved in the gene coding for enzyme families of plants that control various metabolic processes. A similar approach was undertaken by Li et al. (2017a) in *Arabidopsis* species revealed a modulation of anthocyanin accumulation can be achieved by targeting UDP-glycosyltransferases genes that directly contribute to salt, cold, and drought-related stress tolerance. The absence of abnormal expression of UDP-glycosyltransferases genes lead to decreased accumulation of anthocyanin and declined antioxidant production; thus, failing to improve the coping mechanism of stress in these plants. In contrast to previous studies mentioned, although the CRISPR/Cas9 system generated mutants were more susceptible to adverse conditions, the efficient role of ugt79b2/b3 UDP-glycosyltransferases genes was clearly identified for their functional role in *Arabidopsis* species.

### Cas9 Variants and Promoter Correspondence

A recent study by Feng et al. (2018) revealed the importance of the role played by promoters in the CRISPR/Cas9 system of plants. Among the three loci targeted in the maize genome by using *dmc1* gene promoter with the U3 promoter for the sgRNA and Cas9, high efficiency of gene editing was observed in T0 plants with 66% stable transmission of the mutation to the T1 generation. More importantly, re-sequencing of the whole genome revealed zero off-target mutations.

**FIGURE 3:**
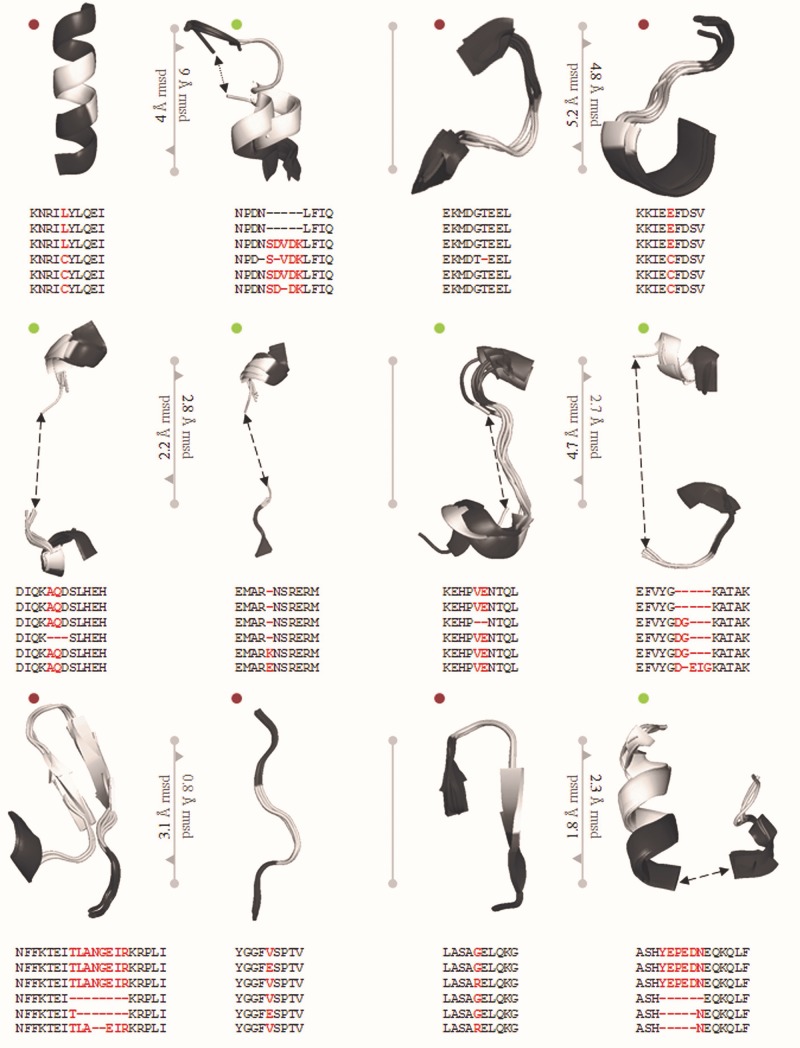
PDB ID of Super positioned structures: 5FW2, 5FW3, 5FW1, 5B2S, 5B2R, and SB2T. The reported mutations are VQR: D1135V, R1335Q and T1337R, EQR: D1135E, R1335Q and T1337R, VRER: D1135V, G1218R, R1335E and T1337R. In total, 12 mutations screened from Anders et al., and Hirano et al., reported structure in which 6 synonymous syntactic parsers from PDB and 6 non-synonymous mutations. sSNP and nsSNP are significant roles in choosing guide RNA specificity. The screened mutated regions are used to choose Cas9 variant for genome editing in crops.

Genome editing by CRISPR/Cas9 system heavily depends on the sequences recognized by Cas9. This recognition has been heavily restricted by the specificity of the PAM. The PAM sequence (5′-NGG-3′) was addressed and altered by studying the structural details to engineer Cas9 derivatives and enable a more vigorous selection of gene sites and improve specificity (Kleinstiver et al., 2015). Engineered sequences obtained from the traditional PAM, recognizes the 5′-NGA-3′, 5′-NGAG-3′, and 5′-NGCG-3′ PAMs for VQR, QR, and VRER and provide better discrimination of off-targets and establish the feasibility of functional efficiency (Figure 3). This engineering based on *S. pyogenes* by Hirano et al. (2016) provides a broad establishment of a framework for rational engineering in CRISPR/Cas9 system. Figure 3 depicts the essentiality of a single nucleotide change that would enhance, hinder, modulate or modify the activity of Cas proteins.

Although studies by Kleinstiver et al. (2015) showed a twofold increase in gene targeting by using VQR, QR, and VRER variants of PAM within the human genome (Table 4), the VQR variant showed a low editing frequency in rice. The robustness of the VQR variant in genome editing was significantly increased by inducing modifications in the structure of sgRNA.

**TABLE 4:** Modified PAM sequences among various variants of SpCas9 and their respective mutations.

**SpCas9 variant**	**Mutations (relative to SpCas9)**	**PAM sequence**
D1135E variant	D1135E	NGG
VQR variant	D1135V, R1335Q and T1337R	NGAN or NGNG
EQR variant	D1135E, R1335Q and T1337R	NGAG
VRER variant	D1135V, G1218R, R1335E and T1337R	NGCG

## Spurt of CRISPR in Crop Improvement

In-depth studies in CRISPR/Cas made an impressive entry to the gene-editing platform to rapidly produce useful novel phenotypes/traits (Baker, 2014; Feng et al., 2014; Gao and Zhao, 2014; Zhang et al., 2014). Primarily, CRISPR/Cas9-based genome editing requires delivery of sgRNA and Cas9 protein into the target cells (Kumar and Jain, 2015). Once this is achieved, CRISPR/Cas9 tools can be adapted for studying plant growth, productivity and development. Other aspects where such a validated genome editing tool can be used is among the study of the metabolic pathways, making the plants resistant to various biotic and abiotic stresses, nutrient uptake, better knowledge of cell cycle and regulation, and successful manipulation of photosynthetic efficiency. A detailed overview of some economically important plants with CRISPR/Cas9 technology as presented in the database www.lipre.org is described below. This library and database created and managed by the Nutritional Improvement of Crops at the International Centre for Genetic Engineering and Biotechnology, New Delhi, India, focuses on both fundamental and applied research worldwide, with special attention to crop specific review and original research articles that streamlines the global contribution of CRISPR based genome editing.

### Fruit Crops

Precise editing was used to achieve an albino phenotype by targeting phytoene desaturase in watermelon (Tian et al., 2017) using protoplast cells. Highly valued crops like *citrus* that face many traditional breeding challenges such as long crossing cycles, extended juvenility, polyploidy and polyembryony were also modified by CRISPR/Cas9/sgRNA technology to yield no side effects and off-target mutated disease resistant plants (Jia et al., 2017a,b). *Citrus* and grape are economically valuable fruits that breeders target numerous fruit quality traits such as fruit size, disease tolerance, abiotic stress tolerance and aroma. Phytoene desaturase gene *CsPDS* and *cpPDS* were targeted by Xcc-filtrated agroinfiltration of SpCas9/sgRNA in sweet orange and *Citrus* paradise (Jia and Wang, 2014; Jia et al., 2017a). Resistance to *citrus* canker was established by targeting regulatory regions of *CsLOB1* gene critical in promoting pustule formation and pathogen growth. Enhanced resistance was observed with a promoter editing that targets the effector binding element in Waijincheng orange (Peng et al., 2017). Similar resistance was observed by inducing mutation in the CDS of both alleles of *CsLOB1* in Duncan grapefruit (Jia et al., 2017b). CRISPR/Cas9 expression achieved an early stage gene editing in *citrus* by targeting phytoene desaturase (PDS) gene using YAO promoter (Zhang et al., 2017).

Interestingly, optimization of CRISPR/Cas system in one fruit crop was also explored for feasibility in another crop of different family. Two different gRNAs associated to the Cas9 of PDS and flowering gene was placed under the control of U3 and U6 apple promoters. Although, the efficiency of transgenes was higher in apple (80%) than pear (9%) for the early flowering gene, the transient transformation of CRISPR-PDS construct produced T-DNA free edited lines. Despite variation in the chimerism and edition frequency, targeted mutagenesis was achieved in the T1 generation of pear and apple lines (Charrier et al., 2019).

No off-target mutations among the regenerated grape plantlets was suggested by using CRISPR/Cas9 to target L-idonate dehydrogenase gene in Chardonnay suspension cells (Ren et al., 2016). Contrarily, increased incidence of DSBs with or without defective repair mechanisms in older leaves was observed on targeted mutagenesis of grape phytoene desaturase (*VvPDS*) (Nakajima et al., 2017). *Vitis vinifera*, the widely cultivated grape variety, showed five types of CRISPR/Cas9 target sites for potential genome editing (Wang et al., 2016). Purified ribonucleoproteins of CRISPR/Cas9 were effective in targeting *MLO-7* and producing resistance to powdery mildew in grape protoplasts (Malnoy et al., 2016). Knockout of transcription factor VvWRKY52 in grape increased fungal resistance against *Botrytis cinerea* as proof for its role in biotic stress response (Wang X. et al., 2018).

### Wheat

Wheat (*Triticum aestivum*) was one of the first model system to corroborate CRISPR/Cas9 technology (Shan et al., 2013). The attempts resulted in generating knockouts of both *PDS* and inositol oxygenase genes (Upadhyay et al., 2013). Interestingly, co-expression of two multiplexed sgRNAs genes targeting two conjointly distributed target sequences in the wheat genome deleted the DNA segment between the two sites. TECCDNA and TECCRNA based on transient expression of CRISPR/Cas9 DNA in wheat plants without herbicide or antibiotic selection was developed by Zhang and co-workers in 2016. Six different genes were validated to establish the effectiveness of the two new methods for generating targeted mutants with no detectable transgene in the T0 generation. The authors have expressed that the same technique can be adapted to vegetative propagated crops such as banana, cassava and potato. Replicon-based system for genome engineering in wheat cells achieved a 110-fold increase in the reporter gene thus making it possible to edit complex cereal genomes using a deconstructed version of viruses (Gil-Humanes et al., 2017). The efficiency of gene targeting was analyzed by a few research groups by using DNA-virus based amplicons using CRISPR/Cas9 cassettes for straightforward and transient expression. Nearly 70% of wheat protoplasts were transfected successfully with wheat dehydration responsive element binding protein 2 (TaDREB2) and wheat ethylene responsive factor 3 (TaERF3) by Kim et al. (2018). The study involving targeting TaGW2 (a negative regulator of grain traits), TaLpx-1 (lipoxygenase, which provides resistance to *Fusarium*) and TaMLO (loss of function, confers resistance to powdery mildew resistance) resulted in a dependable gene editing efficiency in four successive generations (Wang W. et al., 2018). The precision and acceleration in crop improvement by avoiding transgene integration and reduced off-target mutations was reported by Liang et al. (2018) for bread wheat by using CRISPR/Cas9 ribonucleoproteins. Diet based recovery from coeliac disease was confirmed by the generation of low-gluten, transgene-free wheat lines by CRISPR/Cas system by Sánchez-León et al. (2018).

### Rice

Rice is one of the first monocot plant crop species to go through gene editing using TALENs (Li et al., 2012; Shan et al., 2013) and Cas9/gRNA (Feng et al., 2013, Jiang et al., 2013; Weeks et al., 2016). Subsequently, a large number of experiments have been carried out on rice crops as a model to show specificity of Cas9 targeted mutagenesis (Miao et al., 2013; Endo et al., 2015; Xie et al., 2015; Mazumdar et al., 2016; Sun et al., 2016; Weeks et al., 2016). CRISPR/Cas9 system has conferred bi-allelic gene modifications in a single generation, gene replacement through site-specific homologous recombination and opportunity to delete large segments of chromosomes (Feng et al., 2013; Xu et al., 2014; Zhou et al., 2014; Weeks et al., 2016). The positive and negative selection systems available in rice offer a rapid means of generating genetically altered monocots (Shimatani et al., 2015; Weeks et al., 2016). dCas9 fused with cytidine deaminase was used for base editing of herbicidal gene (*C287*) without introduction of DSBs using activation-induced cytidine deaminase (Shimatani et al., 2017). Successful application of base editing using BE3 base editor for *OsPDS* and *OsSBEIIb* target genes that combines the tool of uracil glycosylase inhibitor, nicked Cas9, and cytosine deaminase that inhibits base-excision repair (Li et al., 2017a). Multiplex genome editing in rice and *Arabidopsis* has been confirmed and made into a potential easy technique. This strategy was followed by Shen et al. (2017) by targeting eight genes of agrarian importance using a single binary vector ligated by isocaudamer methodology. CRISPR was combined with QTL editing approach to increase the grain size and grain number in rice varieties (Shen et al., 2018). A-G conversion was introduced successfully in rice using nickase TadA: TadA7.10 heterodimer (Yan et al., 2018). Rice has also been targeted fervently by research groups using CRISPR/Cas-Cpf1 mediated genome editing (Foster et al., 2018; Li et al., 2018b,c; Macovei et al., 2018; Tang et al., 2018; Wang M. et al., 2018; Zhang et al., 2018).

### Maize

In addition to rice being a fodder crop, maize (*Zea mays*) is also a model plant for genetic research. The precise gene editing of several genes in maize has been successfully carried out using CRISPR/Cas9 systems and TALENs (Liang et al., 2014). Specifically, the Cas9/sg-RNA system can be used to generate efficient gene knockouts and gene replacements. Delivery of gene associated or naked sgRNAs expressing Cas9 gene results in a single stranded DNA gene replacement or a DSB in immature embryos of maize (Weeks et al., 2016). Effective strategy using CRISPR resulted in knockout of genes involved in phytic acid synthesis (Liang et al., 2014) and editing of phytoene synthase gene using maize U6 snRNA promoter resulted in white kernels and albino seedlings (Zhu et al., 2016). T0 maize lines showed 31% mutation efficiency using *Agrobacterium* mediated transformation of maize embryos using maize U3 promoter and sgRNAs designed to target knockout of albino marker gene Zmzb7 (Feng et al., 2016). A multiplex editing vector that incorporates a cluster of gRNAs targeting RPL, PPR, and IncRNA increased the editing efficiency up to 100% in maize (Qi et al., 2016). Thermosensitive genic male-sterile 5 (*ZmTMS5*), known to cause male sterility was knocked out in maize protoplasts by CRISPR/Cas9 approach using three gRNAs, with one sgRNA targeting the first exon and the other two sgRNAs targeting the second exon (Li et al., 2017b). Two genome edited variants that utilized the CRISPR/Cas13 approach for overexpression of ARGOS genes were used for the production of hybrids with improved yield under drought (Shi et al., 2017). In addition to Cas9 approaches, Cas9 nickases has been used to provide an advantage for genome modifications in certain loci in target complexes (Wolter et al., 2017). Further, dmc1 gene promotor combined with U3 promoter for Cas9 and sgRNA creates a highly efficient genome editing in maize (Feng et al., 2018). In our laboratory we have designed constructs employing CRISPR/Cas9-based gene editing of native 5-enolpyruvylshikimate-3-phosphate (*EPSP*) synthase gene with two sgRNAs and homology donor repair template. Our research group has obtained 10 out of 20 T_0_ maize lines that showed the introduction of three mutations in native EPSP gene for conferring glyphosate tolerance in maize (Provisional Patent Filed: 201711041380; TEMP/E-1/42049/2017-DEL).

### Soybean

Reports by Jacobs et al. (2015) reflected the use of CRISPR/Cas9 system for soybean. *Agrobacterium rhizogenes*-derived hairy root soybean cultures or somatic embryo cultures derived GFP and nine endogenous loci when targeted by CRISPR/Cas9 system revealed a number of gene editing events that interestingly increased with time (Weeks et al., 2016). Soybean GmU6-16-1 promoter was found to be more efficient in simultaneous editing of multiple homeoalleles relative to the *Arabidopsis* AtU6-26 promoter for HDR-mediated gene integrations at callus stage (Sun et al., 2016). Rj4, the dominant nodulation restriction gene in soybean was validated for the same activity in many strains of *Bradyrhizobium elkanii* using CRISPR/Cas9 strategy and complementation (Tang et al., 2016). Homologous gene replacement of Avr4/6 by a marker gene (NPT II) stimulated by the CRISPR/Cas9 system emphasized gene-based pathogen recognition system by plants containing the soybean R gene loci, Rps4, and Rps6 (Fang and Tyler, 2016). CRISPR knockout of the soybean flowering time gene resulted in homozygous GmFT2a mutants till T2 generation exhibiting late flowering under both long-day and short-day conditions (Cai et al., 2018).

### Tomato

The efficiency in transformation experiments using *Solanum lycopersicum* has proven it to be a perfect dicot candidate for testing CRISPR/Cas9 gene editing (Van Eck et al., 2006). Efficient gene editing of tomato in the first generation using CRISPR/Cas9 systems was demonstrated by generating a wide range of targeted mutations and a suitable size of homozygous deletions by using two sgRNAs in the F1 generation (Brooks et al., 2014). Some reports pointed out the transient use of CRISPR/Cas9 in tomato roots (Brooks et al., 2014; Ron et al., 2014) by *Agrobacterium* mediated transformation. In addition to *Agrobacterium* species, Cermak et al. (2011) showed that the Geminivirus vectors are an efficient mechanism for gene targeting in tomato. Off-targeting gene analysis in tomato can be furthered by using genome sequencing experiments. Florigen paralog and flowering repressors that drive the loss of day-light sensitive flowering when mutated by CRISPR system resulted in rapid flowering, early bursts in flower production, thus favoring a better yield (Soyk et al., 2017). The dominant *ALC* (Alcobaca) was replaced with the recessive *alc* to increase the shelf life of T1 homozygous tomato using HDR-mediated replacement (Yu et al., 2017). Seedless fruit bearing tomato plants generated by somatic mutation of *SIIAA9* revealed changes in leaf shape in addition to the parthenocarpic nature of fruits (Ueta et al., 2017). Soyk et al. (2017) showed CMGE in *SP5G* responsible for tomato flowering repressor improves the architecture of inflorescence and yield of fruit.

One to four gene mutants were generated in 53 genome-edited plants targeting γ-aminobutyric acid (GABA) shunt pathway in tomatoes by CRISPR (Li et al., 2018b). Heritable modifications with a clear albino phenotype was obtained by Pan et al. (2016) by targeting *PDS* in addition to tomato phytochrome interacting factor. Similar strategy was utilized for reshuffling of chromosomal segments in somatic cells of tomato using *PSY1* as a marker gene (Hayut et al., 2017).

## Insights on CRISPR/Cas9 System

Recent research indicates that the CRISPR/Cas9 system has a great perspective for conferring the plant immunity, as it can be used to target the several sites at viral genomes or different viruses in the same plant simultaneously. Variations have been observed in the optimization of Cas9 proteins used in CRISPR systems. Some research groups have used and shown a plant-codon optimized version of Cas9 (Jiang et al., 2013; Li J.F. et al., 2013; Miao et al., 2013; Shan et al., 2013; Kumar and Jain, 2015) and human codon-optimized version as well (Feng et al., 2013; Mao et al., 2013; Nekrasov et al., 2013; Upadhyay et al., 2013; Xie and Yang, 2013). In plants various promoters have been used to drive Cas9 expression (Kumar and Jain, 2015), among which, the CmV35S promoter has been most commonly used for CRISPR assisted breeding in crops by the formation of novel allelic variants (Belhaj et al., 2013; Kumar and Jain, 2015).

CRISPR/Cas9 technology has also been adapted to generate mutant miRNA binding sites (Bassett et al., 2014) and its interference platform (CRISPRi) imparts a complementary approach to RNAi (Larson et al., 2013; Qi et al., 2013; Kumar and Jain, 2015). CRISPR/Cas9 mediated gene editing technology opens a door to human therapeutic applications by generating synthetic proteins translated from or independent of artificial genes associated with transgenic plants (Webber, 2014).

Allied protocols for gene-based analysis such as next-generation sequencing have also been used for increasing the efficiency of the CRISPR/Cas9 system. In a study by Chen et al. (2018), Illumina sequencing with high resolution melting analysis was developed to verify tetra allelic mutants generated without sexual aggregation using the transient system of CRISPR/Cas9 mediated by *Agrobacterium*. 17.2% of the generated *PDS* gene-based mutants was revealed in an overall population that showed 8.2% of non-transgenic mutation rate.

Loss of function mutants generated by targeting long non-coding RNAs by CRISPR/Cas9 induced gene editing in tomatoes revealed repressed production of ethylene and lycopene production, downregulation of carotenoid and ethylene biosynthesis and altered expression of ripening-related genes leading to repressed ripening process leading to fresher tomato fruits obtained from mutant tomato generations (Guo et al., 2017).

In order to analyze the efficiency of CRISPR/Cas9, the system generated a mutation in subsequent generations, continuous induction of mutation in T0 and the advancement of transgenic nature in T1 and T2 *Glycine max* plants was undertaken by Kanazashi et al. (2018). Two peapod loci were simultaneously targeted for site-directed mutagenesis by CRISPR/Cas9 gRNA-based *Agrobacterium*-mediated transformation. Putative mutations induced in the T0 plants were observed in the T1 generation. As the germ cells of T1 generation showed mutations, the valid proof was obtained for the simultaneous site-directed mutagenesis in the T2 generation. Thirty-three percent of the T2 seeds showed a mutation in the GmPPD loci with 19% of double mutants not evidenced with the Cas9 construct.

He et al. (2018) demonstrated the use of CRISPR/Cas9 for revealing the interaction of a subgroup of phosphofructokinases with plastid encoding genes that regulate chloroplast synthesis in rice. The mutations generated created albino varieties of rice and interaction analysis of the same was confirmed by pull down analysis, yeast two-hybrid, and biomolecular fluorescence complementation experiments in addition to qPCR and immunoblotting.

## Operands of CRISPR/Cas Genome Editing Technology in Plants

### Identifying a Target Site in the Genome

A key prerequisite for a targeted gene editing tool is its ability to discriminate between homologous off-target sites and on-target sequences. The practical approach for knockout experiments reveals a great deal of flexibility for identifying target sites as it involves the introduction of the frame shift mutation in the gene that may be achieved without knowing an exact location of the gene. As the result is usually a truncated product of the protein with the shortest length, the first exon that contains coding sequence is generally targeted. *In vitro* and cellular assays have recently improved the characterization of selected guide RNAs by providing essential information that influences the Cas9 nuclease activity, specificity and to identify the seed region of PAM sequence which in turn is critical for recognition of target sequences. This approach is aided by the use of designing algorithms that improves the fidelity of CRISPR. Further truncated gRNAs which are about 18 nucleotides in length reduce off target DSBs significantly; thus, making large scale analysis of proposed modifications plausible (Chakrabarti et al., 2019). In some cases which involves genes with alternative start sites or alternative splicing or exons, this may not work and the selection of the earliest coding exon is favored. For knock-in experiments, the site selection is constrained by the need to place a DSB as close as possible to the site of the variant, ideally less than 10–15 bp.

While identifying the site of the mutation, particularly one that is reported in literature, the use of cDNA (with no presence of introns) is required. However, for design of gRNA, the genomic sequence is required. Use of the cDNA for gRNA designing may not work if the 20 nucleotides of interest are present between the exon-intron junction and this can result in the protospacer inadvertently span across two exons.

### Delivery Efficacy

Delivery for the CRISPR/Cas system can be categorized into two: delivery vehicle and cargo. Three approaches to cargo of the CRISPR tool are commonly reported: (1) Cas9 protein with gRNA; (2) DNA plasmid that encodes both Cas9 protein and gRNA; and (3) mRNA for translation of Cas9 with an independent gRNA. Depending on whether the system is usable under *in vitro* or *in vivo* conditions, these three cargoes are packed according to the considerations of whether the Cas9 protein is positively charges, how controlled the concentration of Cas9 must be, whether the introduction is of the Cas9 DNA or of the protein and how functional should the Cas9 units be when present in the system at any given timepoint. Three general groups have also been used as vehicles to deliver the gene editing cargo and can be classified as non-viral vectors, viral vectors and physical delivery. Although non-viral vectors such as lipid nanoparticles and penetrating peptides are not as prominent as viral-based delivery, they have been found to show demonstrations for CRISPR applications. Viral delivery methods such as engineered or full-size adenoviruses and/or lentiviruses have been found to show an impact on *in vivo* work. For plant biotechnological approaches, however, physical delivery methods such as electroporation, microinjection and hydrodynamic delivery are under investigation (Lino et al., 2018). CRISPR/Cas9 largely depends on the effective delivery of the components into a plant (Basak and Nithin, 2015). For this, NHEJ repair is not a precise mechanism as it generates endogenous gene disruption/mutagenesis by introducing Indel mutations (Lloyd et al., 2005; Belhaj et al., 2015). To overcome this, Baltes et al. (2014) developed an efficient and facile Gemini-virus system that replicates through double-stranded intermediates. Gemini-viruses are circular, ss-DNA, which infects both monocots and dicots and can engineer delivery into a vast range of crops.

### Off-Target Mutations

According to some researchers, off target mutation, also termed as “stray mutations,” can fall below the background mutation frequency using a well-designed nuclease. It is well agreed that gene editing tools that reach the market should not carry the risk of mutation rates (Aryal et al., 2018). Although this gene editing tool is being developed with increased specificity, there have been reports of the DSB and the subsequent generation of off-target mutagenesis. This phenomenon most likely occurs at sites with sequence similarity to the on-target site and can confound experiments. The pitfall of off-targeting in plants is presently addressed to minimize possible impacts by *in silico* approaches (Xie et al., 2014). Several web servers have been developed that allow the input of the target sequence and help search for similar sequences with a small number of mismatches through the genome of interest. This can be helpful to design several gRNAs and select the gRNA that could result in the least off target effects. Additionally, Basak and Nithin (2015) had discussed the following strategies for minimizing off-target mutations that includes the use of highly specific target sequence, truncation of gRNAs, constructions of mutations using Cas9 and a short selection period of calli during regeneration. Recent studies involving the use of whole genome sequencing have confirmed the frequency of off-target mutations may be highly specific depending on the cell type (Veres et al., 2014).

### Stable Inheritance of Phenotypic Variation Through Multiple Generations

Although the advent of CRISPR/Cas9 driven strategies has revolutionized the modulation of gene expression on par with RNAi (previously considered the gold standard for targeted silencing of genes), single or multiple gene targeting can be undertaken at a DNA level with greater specificity. Parallelly using RNA as the platform for transcriptional regulation, Cas13 has been analyzed with increased efficiency for the development of RNA-specific technologies (Gootenberg et al., 2017; Aman et al., 2018). In addition to such advancements, tissue specific promoter systems with tRNAs flanking the desired guide RNAs coupled with a self-cleaving ribozyme provides cell and tissue specificity and the possibility of gRNA expression from any desired promoter. Such Cas9 and Cas13 systems also uncover new possibilities for engineering transcriptomes and modulating gene expression patterns. As the ribosomal loading of transcripts and translation are dependent on the circadian rhythm in plants, the timing of expression if taken into consideration would reap maximum benefits and may even coincide with the wild-type expression contexts (Jabre et al., 2019). We envisage that further refinement of the CRISPR/Cas tool and strategy could be possible by understanding the chromatin context, chromatin language, regulatory framework, and engineered biological network.

### Designing an Effective gRNA

A successful gRNA must maximize on-target activity (guide efficiency) while also minimizing potential off-target effects (guide specificity). Balancing these two requirements can be a combinatorial challenging task and as a result, significant effort in recent years has been focused on developing computational tools to assist in the design of gRNAs. Primarily this effort includes avoiding poly-T sequences, limiting the GC content and a G immediately upstream of the PAM (i.e., an GNGG motif). The upsurge of online tools and software to devise specific and efficient gRNAs reflects the importance of gRNA design as one of the key factors in the CRISPR tool. Researchers from Broad Institute have developed an online tool called CRISPR design to design a single sequence of gRNA or batch mode to predict several gRNAs simultaneously; both of which evaluates off-target effects (Liu et al., 2017)^1^. The same program also assesses mismatches and off-target effects similar to other tools such as CRISPR-P, E-CRISPR, Cas-OFFinder, Cas OT, Cas Designer, and SS Finder (Liu et al., 2017). More recent studies have also begun to include non-sequence information, such as thermodynamic stability of the gRNA and position of the cut site relative to the transcription start site (Wilson et al., 2018).

## Future Perspectives and Prospects for Small RNA

Genome editing holds a significant potential for advancing in elementary knowledge for generating crop plants with effective novel and pertinent nutritional and agronomic, traits for the comforts of consumers and farmers. The huge amount of scientific interest CRISPR has generated is holding its promise as a large number of successful experiments are being carried out in labs from the issue of creating a knockout model of diseases and to help improve nutritional factor of crops. It holds promise in diverse arrays of fields but with it comes complications of ethical issues, misuse and uncertainty over the extent to which it can be used to change the way we look at and manipulate nature itself. The scope of the CRISPR toolkit is applicable to a wide array of possibilities ranging from gene disruption, gene knockout, and promoter study and conditional knockout analyses. More recently Zhao et al. (2016) proposed an alternative approach by using a dual-sgRNA/Cas9 design where the first construct was used to successfully integrate with the miRNA (*MIR169a* and *MIR827a* loci) and the second construct for HDR corresponding to gRNAs was introduced. Although the efficiency of this transfer was 0.8% in four of 500 T0 plants, this successful establishment of gene deletion in stable lines pave the way for introduction og genes of interest for targeted crop improvement.

## Conclusion

A PubMed search for the term “CRISPR” performed on May 18, 2019 showed a total of 13961 hits with a total of 2713 contributions till the year 2018 alone; which includes nearly 80% of the contributions in 2017. Although only 1671 contribute to the use of CRISPR toolkit in plants, it is a safe estimation that this number will be much higher and there will be many new breakthroughs by the time this paper is published. Being an easy and economical tool, CRISPR assures reform in basic and applied research and promote the application of developed technology in agriculture. The challenges associated with transformation protocols, crop specific vectors and genome resources can be handled along with the continued evolution of the CRISPR system and improvements of CRISPR components.

## Author Contributions

TK, NMR, and ME were involved in the content of the manuscript and its design. AT, RV, KMS, RK, and SKS helped with editing the manuscript. PY and PKA were involved in providing technical comments.

## Conflict of Interest Statement

The authors declare that the research was conducted in the absence of any commercial or financial relationships that could be construed as a potential conflict of interest.

## Footnotes

1 www.genome-engineering.org

## Supplementary Material

MATERIAL S1 CRISPR Edited gene lists. List Genes edited in the crops for various functional features which includes PMD ID, Gene Name, PMID, Article Title, Abstract, First Author (First Last), All authors (Last, Initials), Affiliations.

MATERIAL S2 Timeline data fragments of Whole, Review and Original Articles; Sheet 1: Composites of all CRIPSR Specific articles with PMID, Digital Object ID, Article Title, Abstract, Language, Journal, Title Journal, Abbreviation Journal, NLM ID, Journal Year, Journal Month, Journal Medline Date, Journal Volume, Journal Issue, Journal Medline Pagination, Citation Medium, First Author (First Last), All authors (Last, Initials), Affiliation, Related, Citations, Related Citation, Related Articles by Review, Related Articles by Review (Medline format), Article Type.

MATERIAL S3 Vector annotation profiles from published citations. Annotation profile includes Vector Name, Feature names, Type of features, Sequence, Minimum length, Maximum Length, Gap Intervals, Direction-Forward/Reverse.

## References

[B1] Abudayyeh O. O. Gootenberg J. S. Konermann S. Joung J. Slaymaker I. M. Cox D. B. (2016). C2C2 is a single-component programmable RNA-guided RNA-targeting CRISPR effector. *Science* 353:aaf5573. 10.1126/science.aaf5573 27256883 PMC5127784

[B2] Aman R. Ali Z. Butt H. Mahas A. Aljedaani F. Khan M. Z. (2018). RNA virus interference via CRISPR/Cas13a system in plants. *Genome Biol.* 19:1. 10.1186/s13059-017-1381-1 29301551 PMC5755456

[B3] Anantharaman V. Makarova K. S. Burroughs A. M. Koonin E. V. Aravind L. (2013). Comprehensive analysis of the HEPN superfamily: identification of novel roles in intra-genomic conflicts, defense, pathogenesis and RNA processing. *Biol. Direct.* 8:15. 10.1186/1745-6150-8-15 23768067 PMC3710099

[B4] Anders C. Niewoehner O. Duerst A. Jinek M. (2014). Structural basis of PAM-dependent target DNA recognition by the Cas9 endonuclease. *Nature* 513 569–573. 10.1038/nature13579 25079318 PMC4176945

[B5] Aparicio-Prat E. Arnan C. Sala I. Bosch N. Guigó R. Johnson R. (2015). DECKO: single-oligo, dual-CRISPR deletion of genomic elements including long non-coding RNAs. *BMC Genomics* 16:846. 10.1186/s12864-015-2086-z 26493208 PMC4619085

[B6] Aryal N. K. Wasylishen A. R. Lozano G. (2018). CRISPR/Cas9 can mediate high-efficiency off-target mutations in mice in vivo. *Cell Death Dis.* 9:1099. 10.1038/s41419-018-1146-0 PMC6204134 30368519

[B7] Attar N. (2015). Techniques & applications: Cpf1 makes for a CRISPR cut. *Nat. Rev. Microbiol.* 13:660.

[B8] Baker M. (2014). Gene editing at CRISPR speed. *Nat. Biotechnol.* 32 309–312. 10.1038/nbt.2863 24714470

[B9] Baltes N. J. Gil-Humanes J. Cermak T. Atkins P. A. Voytas D. F. (2014). DNA replicons for plant genome engineering. *Plant Cell* 26 151–163. 10.1105/tpc.113.119792 24443519 PMC3963565

[B10] Barrangou R. Fremaux C. Deveau H. Richards M. Boyaval P. Moineau S. (2007). CRISPR provides acquired resistance against viruses in prokaryotes. *Science* 315 1709–1712. 10.1126/science.1138140 17379808

[B11] Basak J. Nithin C. (2015). Targeting non-coding RNAs in plants with the CRISPR/Cas technology is a challenge yet worth accepting. *Front. Plant Sci.* 6:1001. 10.3389/fpls.2015.01001 26635829 PMC4652605

[B12] Bassett A. R. Azzam G. Wheatley L. Tibbit C. Rajakumar T. McGowan S. (2014). Understanding functional miRNA-target interactions in vivo by site-specific genome engineering. *Nat. Commun.* 5:4640. 10.1038/ncomms5640 25135198 PMC4143950

[B13] Belhaj K. Chaparro-Garcia A. Kamoun S. Patron N. J. Nekrasov V. (2015). Editing plant genomes with CRISPR/Cas9. *Curr. Opin. Biotechnol.* 32 76–84. 10.1016/j.copbio.2014.11.007 25437637

[B14] Belhaj K. Garcia A. C. Kamoun S. Nekrasov V. (2013). Plant genome editing made easy: targeted mutagenesis in model and crop plants using the CRISPR/Cas system. *Plant Methods* 9:39. 10.1186/1746-4811-9-39 24112467 PMC3852272

[B15] Benda C. Ebert J. Scheltema R. A. Schiller H. B. Baumgärtner M. Bonneau F. (2014). Structural model of a CRISPR RNA-silencing complex reveals the RNA-target cleavage activity in Cmr4. *Mol. Cell.* 56 43–54. 10.1016/j.molcel.2014.09.002 25280103

[B16] Bertier L. D. Ron M. Huo H. Bradford K. J. Britt A. B. Michelmore R. W. (2018). High-resolution analysis of the efficiency, heritability, and editing outcomes of CRISPR/Cas9-induced modifications of NCED4 in lettuce (*Lactuca sativa*). *G3 (Bethesda).* 8 1513–1521. 10.1534/g3.117.300396 29511025 PMC5940144

[B17] Bhavya D. Davison M. Barrangou R. (2011). CRISPR/Cas systems in bacteria and archaea: versatile small RNAs for adaptive defense and regulation. *Annu. Rev. Genet.* 45 273–297. 10.1146/annurev-genet-110410-132430 22060043

[B18] Bolotin A. Quinquis B. Sorokin A. Ehrlich S. D. (2005). Clustered regularly interspaced short palindrome repeats (CRISPRs) have spacers of extrachromosomal origin. *Microbiology* 151 2551–2561. 10.1099/mic.0.28048-0 16079334

[B19] Brooks C. Nekrasov V. Lippman Z. B. Van Eck J. (2014). Efficient gene editing in tomato in the first generation using the clustered regularly interspaced short palindromic repeats/CRISPR-associated9 system. *Plant Physiol.* 166 1292–1297. 10.1104/pp.114.247577 25225186 PMC4226363

[B20] Brouns S. J. Jore M. M. Lundgren M. Westra E. R. Slijkhuis R. J. Snijders A. P. (2008). Small CRISPR RNAs guide antiviral defense in prokaryotes. *Science* 321 960–964. 10.1126/science.1159689 18703739 PMC5898235

[B21] Cai Y. Chen L. Liu X. Guo C. Sun S. Wu C. (2018). CRISPR/Cas9-mediated targeted mutagenesis of GmFT2a delays flowering time in soya bean. *Plant Biotech. J.* 16 176–185. 10.1111/pbi.12758 28509421 PMC5785355

[B22] Cermak T. Doyle E. L. Christian M. Wang L. Zhang Y. Schmidt C. (2011). Efficient design and assembly of custom TALEN and other TAL effector-based constructs for DNA targeting. *Nucleic Acids Res.* 39:e82. 10.1093/nar/gkr218 21493687 PMC3130291

[B23] Chakrabarti A. M. Henser-Brownhill T. Monserrat J. Poetsch A. R. Luscombe N. M. Scaffidi P. (2019). Target-specific precision of CRISPR-mediated genome editing. *Mol. Cell.* 73 699–713. 10.1016/j.molcel.2018.11.031 30554945 PMC6395888

[B24] Charrier A. Vergne E. Dousset N. Richer A. Petiteau A. Chevreau E. (2019). Efficient targeted mutagenesis in apple and first time edition of pear using the CRISPR-Cas9 system. *Front. Plant Sci.* 10:40. 10.3389/fpls.2019.00040 30787936 PMC6373458

[B25] Chen X. Xu F. Zhu C. Ji J. Zhou X. Feng X. (2014). Dual sgRNA-directed gene knockout using CRISPR/Cas9 technology in *Caenorhabditis elegans*. *Sci. Rep.* 4:7581. 10.1038/srep07581 25531445 PMC4273605

[B26] Chen J. S. Ma E. Harrington L. B. Da Costa M. Tian X. Palefsky J. M. (2018). CRISPR-Cas12a target binding unleashes indiscriminate single-stranded DNase activity. *Science* 360 436–439. 10.1126/science.aar6245 29449511 PMC6628903

[B27] Cho S. W. Kim S. Kim Y. Kweon J. Kim H. S. Bae S. (2014). Analysis of off-target effects of CRISPR/Cas-derived RNA-guided endonucleases and nickases. *Genome Res.* 24 132–141. 10.1101/gr.162339.113 24253446 PMC3875854

[B28] Christian M. Qi Y. Zhang Y. Voytas D. F. (2013). Targeted mutagenesis of Arabidopsis thaliana using engineered TAL effector nucleases. *G3* 3 1697–1705. 10.1534/g3.113.007104 23979944 PMC3789794

[B29] Curtin S. J. Xiong Y. Michno J. M. Campbell B. W. Stec A. O. Čermák T. (2018). Crispr/cas9 and TALENs generate heritable mutations for genes involved in small rna processing of glycine max and medicago truncatula. *Plant Biotechnol. J.* 16 1125–1137. 10.1111/pbi.12857 29087011 PMC5978873

[B30] Deltcheva E. Chylinski K. Sharma C. M. Gonzales K. Chao Y. Pirzada Z. A. (2011). CRISPR RNA maturation by trans-encoded small RNA and host factor RNase III. *Nature* 471 602–607. 10.1038/nature09886 21455174 PMC3070239

[B31] Derrien T. Johnson R. Bussotti G. Tanzer A. Djebali S. Tilgner H. (2012). The GENCODE v7 catalog of human long noncoding RNAs: analysis of their gene structure, evolution, and expression. *Genome Res.* 22 1775–1789. 10.1101/gr.132159.111 22955988 PMC3431493

[B32] Deveau H. Barrangou R. Garneau J. E. Labonté J. Fremaux C. Boyaval P. (2008). Phage response to CRISPR-encoded resistance in *Streptococcus thermophilus*. *J. Bacteriol.* 190 1390–1400. 10.1128/jb.01412-07 18065545 PMC2238228

[B33] Durr J. Papareddy R. Nakajima K. Gutierrez-Marcos J. (2018). Highly efficient heritable targeted deletions of gene clusters and non-coding regulatory regions in Arabidopsis using CRISPR/Cas9. *Sci. Rep.* 8:4443. 10.1038/s41598-018-22667-1 29535386 PMC5849686

[B34] Endo M. Mikami M. Toki S. (2015). Multigene knockout utilizing off-target mutations of the CRISPR/Cas9 system in rice. *Plant Cell Physiol.* 56 41–47. 10.1093/pcp/pcu154 25392068 PMC4301742

[B35] Fang Y. Tyler B. M. (2016). Efficient disruption and replacement of an effector gene in the oomycete P hytophthora sojae using CRISPR/C as9. *Mol. Plant Pathol.* 17 127–139. 10.1111/mpp.12318 26507366 PMC6638440

[B36] Feng C. Su H. Bai H. Wang R. Liu Y. Guo X. (2018). High-efficiency genome editing using a dmc1 promoter-controlled CRISPR/Cas9 system in maize. *Plant Biotechnol. J.* 16 1848–1857. 10.1111/pbi.12920 29569825 PMC6181213

[B37] Feng C. Yuan J. Wang R. Liu Y. Birchler J. A. Han F. (2016). Efficient targeted genome modification in maize using CRISPR/Cas9 system. *J. Genet. Genome* 43 37–43. 10.1016/j.jgg.2015.10.002 26842992

[B38] Feng Z. Mao Y. Xu N. Zhang B. Wei P. Yang D. L. (2014). Multi generation analysis reveals the inheritance, specificity, and patterns of CRISPR/Cas-induced gene modifications in Arabidopsis. *Proc. Natl. Acad. Sci. U.S.A.* 111 4632–4637. 10.1073/pnas.1400822111 24550464 PMC3970504

[B39] Feng Z. Zhang B. Ding W. Liu X. Yang D. L. Wei P. (2013). Efficient genome editing in plants using a CRISPR/Cas system. *Cell Res.* 23 1229–1232. 23958582 10.1038/cr.2013.114 PMC3790235

[B40] Foster A. J. Martin-Urdiroz M. Yan X. Wright S. Soanes D. M. Talbot N. J. (2018). CRISPR-Cas9 ribonucleoprotein-mediated co-editing and counterselection in the rice blast fungus. *bioRxiv* 349134. 10.1038/s41598-018-32702-w 30254203 PMC6156577

[B41] Gao F. Shen X. Z. Jiang F. Wu Y. Han C. (2016). DNA-guided genome editing using the Natronobacterium gregoryi Argonaute. *Nat. Biotechnol.* 34 768–773. 10.1038/nbt.3547 27136078

[B42] Gao Y. Zhao Y. (2014). Self-processing of ribozyme-flanked RNAs into guide RNAs in vitro and in vivo for CRISPR-mediated genome editing. *J. Integr. Plant Biol.* 56 343–349. 10.1111/jipb.12152 24373158

[B43] Garneau J. E. Dupuis M. È. Villion M. Romero D. A. Barrangou R. Boyaval P. (2010). The CRISPR/Cas bacteria immune system cleaves bacteriophage and plasmid DNA. *Nature* 468 67–71. 10.1038/nature09523 21048762

[B44] Gilbert L. A. Larson M. H. Morsut L. Liu Z. Brar G. A. Torres S. E. (2013). CRISPR-mediated modular RNA-guided regulation of transcription in eukaryotes. *Cell* 154 442–451. 10.1016/j.cell.2013.06.044 23849981 PMC3770145

[B45] Gil-Humanes J. Wang Y. Liang Z. Shan Q. Ozuna C. V. Sánchez-León S. (2017). High-efficiency gene targeting in hexaploid wheat using DNA replicons and CRISPR/Cas9. *Plant J.* 89 1251–1262. 10.1111/tpj.13446 27943461 PMC8439346

[B46] Gootenberg J. S. Abudayyeh O. O. Kellner M. J. Joung J. Collins J. J. Zhang F. (2018). Multiplexed and portable nucleic acid detection platform with Cas13, Cas12a, and Csm6. *Science* 360 439–444. 10.1126/science.aaq0179 29449508 PMC5961727

[B47] Gootenberg J. S. Abudayyeh O. O. Lee J. W. Essletzbichler P. Dy A. J. Joung J. (2017). Nucleic acid detection with CRISPR-Cas13a/ C2c2. *Science* 356 438–442. 10.1126/science.aam9321 28408723 PMC5526198

[B48] Guo J. E. Hu Z. Zhu M. Li F. Zhu Z. Lu Y. (2017). The tomato histone deacetylase SlHDA1 contributes to the repression of fruit ripening and carotenoid accumulation. *Sci. Rep.* 7:7930. 10.1038/s41598-017-08512-x 28801625 PMC5554242

[B49] Hayut S. F. Bessudo C. M. Levy A. A. (2017). Targeted recombination between homologous chromosomes for precise breeding in tomato. *Nat. Commun.* 8:15605. 10.1038/ncomms15605 28548094 PMC5458649

[B50] He F. Bhoobalan-Chitty Y. Van L. B. Kjeldsen A. L. Dedola M. Makarova K. S. (2018). Anti-CRISPR proteins encoded by archaeal lytic viruses inhibit subtype I-D immunity. *Nat. Microbiol.* 3:461. 10.1038/s41564-018-0120-z 29507349 PMC11249088

[B51] Hirano H. Gootenberg J. S. Horii T. Abudayyeh O. O. Kimura M. Hsu P. D. (2016). Structure and engineering of *Francisella novicida* Cas9. *Cell* 164 950–961. 10.1016/j.cell.2016.01.039 26875867 PMC4899972

[B52] Ho T. T. Zhou N. Huang J. Koirala P. Xu M. Fung R. (2015). Targeting non-coding RNAs with the CRISPR/Cas9 system in human cell lines. *Nucleic Acids Res.* 43:e17. 10.1093/nar/gku1198 25414344 PMC4330338

[B53] Horvath P. Barrangou R. (2010). CRISPR/Cas, the immune system of bacteria and archaea. *Science* 327 167–170. 10.1126/science.1179555 20056882

[B54] Horvath P. Romero D. A. Coûté-Monvoisin A. C. Richards M. Deveau H. Moineau S. (2008). Diversity, activity, and evolution of CRISPR loci in *Streptococcus thermophilus*. *J. Bacteriol.* 190 1401–1412. 10.1128/jb.01415-07 18065539 PMC2238196

[B55] Hu X. Meng X. Liu Q. Li J. Wang K. (2018). Increasing the efficiency of CRISPR-Cas9-VQR precise genome editing in rice. *Plant Biotechnol. J.* 16 292–297. 10.1111/pbi.12771 28605576 PMC5785341

[B56] Hwang W. Y. Fu Y. Reyon D. Maeder M. L. Kaini P. Sander J. D. (2013). Heritable and precise zebrafish genome editing using a CRISPR-Cas system. *PLoS One* 8:e68708. 10.1371/journal.pone.0068708 23874735 PMC3706373

[B57] Ishino Y. Shinagawa H. Makino K. Amemura M. Nakata A. (1987). Nucleotide sequence of the iap gene, responsible for alkaline phosphatase isozyme conversion in *Escherichia coli*, and identification of the gene product. *J. Bacteriol.* 169 5429–5433. 10.1128/jb.169.12.5429-5433.1987 3316184 PMC213968

[B58] Jabre I. Reddy A. S. N. Kalyna M. Chaudhary S. Khokhar W. Byrne L. J. (2019). Does co-transcriptional regulation of alternative splicing mediate plant stress responses? *Nucleic Acids Res.* 47 2716–2726. 10.1093/nar/gkz121 30793202 PMC6451118

[B59] Jacobs T. B. LaFayette P. R. Schmitz R. J. Parrott W. A. (2015). Targeted genome modifications in soybean with CRISPR/Cas9. *BMC Biotechnol.* 15:16. 10.1186/s12896-015-0131-2 25879861 PMC4365529

[B60] Jia H. Wang N. (2014). Targeted genome editing of sweet orange using Cas9/sgRNA. *PLoS One* 9:e93806. 10.1371/journal.pone.0093806 24710347 PMC3977896

[B61] Jia H. Xu J. Orboviæ V. Zhang Y. Wang N. (2017a). Editing citrus genome via SaCas9/sgRNA system. *Front. Plant Sci.* 8:2135. 10.3389/fpls.2017.02135 29312390 PMC5732962

[B62] Jia H. Zhang Y. Orboviæ V. Xu J. White F. F. Jones J. B. (2017b). Genome editing of the disease susceptibility gene Cs LOB 1 in citrus confers resistance to citrus canker. *Plant Biotechnol. J.* 15 817–823. 10.1111/pbi.12677 27936512 PMC5466436

[B63] Jiang W. Zhou H. Bi H. Fromm M. Yang B. Weeks D. P. (2013). Demonstration of CRISPR/Cas9/sgRNA-mediated targeted gene modification in Arabidopsis, tobacco, sorghum and rice. *Nucleic Acids Res.* 41:e188. 10.1093/nar/gkt780 23999092 PMC3814374

[B64] Jinek M. Chylinski K. Fonfara I. Hauer M. Doudna J. A. Charpentier E. (2012). A programmable dual-RNA-guided DNA endonuclease in adaptive bacterial immunity. *Science* 337 816–821. 10.1126/science.1225829 22745249 PMC6286148

[B65] Jinek M. Jiang F. Taylor D. W. Sternberg S. H. Kaya E. Ma E. (2014). Structures of Cas9 endonucleases reveal RNA-mediated conformational activation. *Science* 343:1247997. 10.1126/science.1247997 24505130 PMC4184034

[B66] Kanazashi Y. Hirose A. Takahashi I. Mikami M. Endo M. Hirose S. (2018). Simultaneous site-directed mutagenesis of duplicated loci in soybean using a single guide RNA. *Plant Cell Rep.* 37 553–563. 10.1007/s00299-018-2251-3 29333573

[B67] Karginov F. V. Hannon G. J. (2010). The CRISPR system: small RNA-guided defense in bacteria and archaea. *Mol. Cell.* 37 7–19. 10.1016/j.molcel.2009.12.033 20129051 PMC2819186

[B68] Kao P. H. Ng I. S. (2017). CRISPRi mediated phosphoenolpyruvate carboxylase regulation to enhance the production of lipid in Chlamydomonas reinhardtii. *Bioresour. Technol.* 245 1527–1537. 10.1016/j.biortech.2017.04.111 28501380

[B69] Kim D. Alptekin B. Budak H. (2018). CRISPR/Cas9 genome editing in wheat. *Func. Integr. Genomics* 18 31–41. 10.1007/s10142-017-0572-x 28918562

[B70] Kim H. Kim J. S. (2014). A guide to genome engineering with programmable nucleases. *Nat. Rev. Genet.* 15 321–334. 10.1038/nrg3686 24690881

[B71] Kim D. Kim S. Kim S. Park J. Kim J. S. (2016). Genome-wide target specificities of CRISPR-Cas9 nucleases revealed by multiplex Digenome-seq. *Genome Res.* 26 406–415. 10.1101/gr.199588.115 26786045 PMC4772022

[B72] Kim J. S. (2018). Precision genome engineering through adenine and cytosine base editing. *Nat. Plants* 4 148–151. 10.1038/s41477-018-0115-z 29483683

[B73] Kleinstiver B. P. Prew M. S. Tsai S. Q. Topkar V. V. Nguyen N. T. Zheng Z. (2015). Engineered CRISPR-Cas9 nucleases with altered PAM specificities. *Nature* 523 481–485. 10.1038/nature14592 26098369 PMC4540238

[B74] Komor A. C. Kim Y. B. Packer M. S. Zuris J. A. Liu D. R. (2016). Programmable editing of a target base in genomic DNA without double-stranded DNA cleavage. *Nature* 533 420–424. 10.1038/nature17946 27096365 PMC4873371

[B75] Kumar V. Jain M. (2015). The CRISPR/Cas system for plant genome editing: advances and opportunities. *J. Exp. Bot.* 66 47–57. 10.1093/jxb/eru429 25371501

[B76] Larson M. H. Gilbert L. A. Wang X. Lim W. A. Weissman J. S. (2013). CRISPR interference (CRISPRi) for sequence-specific control of gene expression. *Nat. Protoc.* 8 2180–2196. 10.1038/nprot.2013.132 24136345 PMC3922765

[B77] LeBlanc C. Zhang F. Mendez J. Lozano Y. Chatpar K. Irish V. F. (2018). Increased efficiency of targeted mutagenesis by CRISPR/Cas9 in plants using heat stress. *Plant J.* 93 377–386. 10.1111/tpj.13782 29161464

[B78] Li C. Zong Y. Wang Y. Jin S. Zhang D. Song Q. (2018a). Expanded base editing in rice and wheat using a Cas9-adenosine deaminase fusion. *Genome Biol.* 19:59. 10.1186/s13059-018-1443-z 29807545 PMC5972399

[B79] Li S. Li J. Zhang J. Du W. Fu J. Sutar S. (2018b). Synthesis-dependent repair of Cpf1-induced double strand DNA breaks enables targeted gene replacement in rice. *J. Exp. Bot.* 69 4715–4721. 10.1093/jxb/ery245 29955893 PMC6137971

[B80] Li S. Zhang X. Wang W. Guo X. Wu Z. Du W. (2018c). Expanding the scope of CRISPR/Cpf1-mediated genome editing in rice. *Mol. Plant* 11 995–998. 10.1016/j.molp.2018.03.009 29567453

[B81] Li R. Li R. Li X. Fu D. Zhu B. Tian H. (2018d). Multiplexed CRISPR/Cas9-mediated metabolic engineering of γ-aminobutyric acid levels in Solanum lycopersicum. *Plant Biotechnol. J.* 16 415–427. 10.1111/pbi.12781 28640983 PMC5787826

[B82] Li D. Qiu Z. Shao Y. Chen Y. Guan Y. Liu M. (2013). Heritable gene targeting in the mouse and rat using a CRISPR/Cas system. *Nat. Biotechnol.* 31 681–683. 10.1038/nbt.2661 23929336

[B83] Li J. F. Norville J. E. Aach J. McCormack M. Zhang D. Bush J. (2013). Multiplex and homologous recombination-mediated genome editing in Arabidopsis and *Nicotiana benthamiana* using guide RNA and Cas9. *Nat. Biotechnol.* 31 688–691. 10.1038/nbt.2654 23929339 PMC4078740

[B84] Li J. Sun Y. Du J. Zhao Y. Xia L. (2017a). Generation of targeted point mutations in rice by a modified CRISPR/Cas9 system. *Mol. Plant* 10 526–529. 10.1016/j.molp.2016.12.001 27940306

[B85] Li J. Zhang H. Si X. Tian Y. Chen K. Liu J. (2017b). Generation of thermosensitive male-sterile maize by targeted knockout of the ZmTMS5 gene. *J. Genet. Genomics* 44 465–468. 10.1016/j.jgg.2017.02.002 28412227

[B86] Li T. Huang S. Jiang W. Z. Wright D. Spalding M. H. Weeks D. P. (2011). TAL nucleases (TALNs): hybrid proteins composed of TAL effectors and FokI DNA-cleavage domain. *Nucleic Acids Res.* 39 359–372. 10.1093/nar/gkq704 20699274 PMC3017587

[B87] Li T. Liu B. Spalding M. H. Weeks D. P. Yang B. (2012). High-efficiency TALEN-based gene editing produces disease-resistant rice. *Nat. Biotechnol.* 30 390–392. 10.1038/nbt.2199 22565958

[B88] Liang Z. Chen K. Zhang Y. Liu J. Yin K. Qiu J. L. (2018). Genome editing of bread wheat using biolistic delivery of CRISPR/Cas9 in vitro transcripts or ribonucleoproteins. *Nat. Prot.* 13 413–430. 10.1038/nprot.2017.145 29388938

[B89] Liang Z. Zhang K. Chen K. Gao C. (2014). Targeted mutagenesis in Zea mays using TALENs and the CRISPR/Cas system. *J. Genet. Genomics* 41 63–68. 10.1016/j.jgg.2013.12.001 24576457

[B90] Lino C. A. Harper J. C. Carney J. P. Timlin J. A. (2018). Delivering CRISPR: a review of the challenges and approaches. *Drug Deliv.* 25 1234–1257. 10.1080/10717544.2018.1474964 29801422 PMC6058482

[B91] Liu X. Wu S. Xu J. Sui C. Wei J. (2017). Application of CRISPR/Cas9 in plant biology. *Acta Pharm. Sin. B* 7 292–302. 10.1016/j.apsb.2017.01.002 28589077 PMC5443236

[B92] Lloyd A. Plaisier C. L. Carroll D. Drews G. N. (2005). Targeted mutagenesis using zinc-finger nucleases in Arabidopsis. *Proc. Natl. Acad. Sci. U.S.A.* 102 2232–2237. 15677315 10.1073/pnas.0409339102 PMC548540

[B93] Lowder L. G. Zhou J. Zhang Y. Malzahn A. Zhong Z. Hsieh T. F. (2018). Robust Transcriptional Activation in Plants Using Multiplexed CRISPR-Act2.0 and mTALE-Act Systems. *Mol. Plant* 11 245–256. 10.1016/j.molp.2017.11.010 29197638

[B94] Lor V. S. Starker C. G. Voytas D. F. Weiss D. Olszewski N. E. (2014). Targeted mutagenesis of the tomato PROCERA gene using transcription activator-like effector nucleases. *Plant Physiol.* 166 1288–1291. 10.1104/pp.114.247593 25217528 PMC4226374

[B95] Lu Y. Zhu J. K. (2017). Precise editing of a target base in the rice genome using a modified CRISPR/Cas9 system. *Mol. Plant* 10 523–525. 10.1016/j.molp.2016.11.013 27932049

[B96] Macovei A. Sevilla N. R. Cantos C. Jonson G. B. Slamet-Loedin I. Čermák T. (2018). Novel alleles of rice eIF4G generated by CRISPR/Cas9-targeted mutagenesis confer resistance to Rice tungro spherical virus. *Plant Biotechnol. J.* 16 1918–1927. 10.1111/pbi.12927 29604159 PMC6181218

[B97] Mahfouz M. M. Piatek A. Stewart C. N. Jr. (2014). Genome engineering via TALENs and CRISPR/Cas9 systems: challenges and perspectives. *Plant Biotechnol. J.* 12 1006–1014. 10.1111/pbi.12256 25250853

[B98] Makarova K. S. Aravind L. Wolf Y. I. Koonin E. V. (2011a). Unification of Cas protein families and a simple scenario for the origin and evolution of CRISPR/Cas systems. *Biol. Direct.* 6:38. 10.1186/1745-6150-6-38 21756346 PMC3150331

[B99] Makarova K. S. Haft D. H. Barrangou R. Brouns S. J. J. Charpentier E. Horvath P. (2011b). Evolution and classification of the CRISPR/Cas systems. *Nat. Rev. Microbiol.* 9 467–477. 21552286 10.1038/nrmicro2577 PMC3380444

[B100] Malina A. Cameron C. J. Robert F. Blanchette M. Dostie J. Pelletier J. (2015). PAM multiplicity marks genomic target sites as inhibitory to CRISPR-Cas9 editing. *Nat. Commun.* 6:10124. 10.1038/ncomms10124 26644285 PMC4686818

[B101] Malnoy M. Viola R. Jung M. H. Koo O. J. Kim S. Kim J. S. (2016). DNA-free genetically edited grapevine and apple protoplast using CRISPR/Cas9 ribonucleoproteins. *Front. Plant Sci.* 7:1904. 10.3389/fpls.2016.01904 28066464 PMC5170842

[B102] Mao Y. Zhang H. Xu N. Zhang B. Gou F. Zhu J. K. (2013). Application of the CRISPR–Cas system for efficient genome engineering in plants. *Mol. Plant.* 6 2008–2011. 10.1093/mp/sst121 23963532 PMC3916745

[B103] Marraffini L. A. Sontheimer E. J. (2008). CRISPR interference limits horizontal gene transfer in staphylococci by targeting DNA. *Science* 322 1843–1845. 10.1126/science.1165771 19095942 PMC2695655

[B104] Marraffini L. A. Sontheimer E. J. (2010). CRISPR interference: RNA-directed adaptive immunity in bacteria and archaea. *Nat. Rev. Genet.* 11 181–190. 10.1038/nrg2749 20125085 PMC2928866

[B105] Mazumdar S. Quick P. W. Bandyopadhyay A. (2016). CRISPR/Cas9 mediated genome editing in rice, advancements and future possibilities. *Ind. J. Plant Physiol.* 21 437–445. 10.1007/s40502-016-0252-1

[B106] Miao J. Guo D. Zhang J. Huang Q. Qin G. Zhang X. (2013). Targeted mutagenesis in rice using CRISPR–Cas system. *Cell Res.* 23 1233–1236. 23999856 10.1038/cr.2013.123 PMC3790239

[B107] Mojica F. J. García-Martínez J. Soria E. (2005). Intervening sequences of regularly spaced prokaryotic repeats derive from foreign genetic elements. *J. Mol. Evol.* 60 174–182. 10.1007/s00239-004-0046-3 15791728

[B108] Nakajima I. Ban Y. Azuma A. Onoue N. Moriguchi T. Yamamoto T. (2017). CRISPR/Cas9-mediated targeted mutagenesis in grape. *PLoS One* 12:e0177966. 10.1371/journal.pone.0177966 28542349 PMC5436839

[B109] Nekrasov V. Staskawicz B. Weigel D. Jones J. D. Kamoun S. (2013). Targeted mutagenesis in the model plant *Nicotiana benthamiana* using Cas9-guided endonuclease. *Nat. Biotechnol.* 31 691–693. 10.1038/nbt.2655 23929340

[B110] Nishida K. Arazoe T. Yachie N. Banno S. Kakimoto M. Tabata M. (2016). Targeted nucleotide editing using hybrid prokaryotic and vertebrate adaptive immune systems. *Science* 353:aaf8729. 10.1126/science.aaf8729 27492474

[B111] Nishimasu H. Ran F. A. Hsu P. D. Konermann S. Shehata S. I. Dohmae N. (2014). Crystal structure of Cas9 in complex with guide RNA and target DNA. *Cell* 156 935–949. 10.1016/j.cell.2014.02.001 24529477 PMC4139937

[B112] Pan C. Ye L. Qin L. Liu X. He Y. Wang J. (2016). CRISPR/Cas9-mediated efficient and heritable targeted mutagenesis in tomato plants in the first and later generations. *Sci. Rep.* 6:24765. 10.1038/srep24765 27097775 PMC4838866

[B113] Peng A. Chen S. Lei T. Xu L. He Y. Wu L. (2017). Engineering canker-resistant plants through CRISPR/Cas9-targeted editing of the susceptibility gene Cs LOB 1 promoter in citrus. *Plant Biotechnol. J.* 15 1509–1519. 10.1111/pbi.12733 28371200 PMC5698050

[B114] Porteus M. H. Carroll D. (2005). Gene targeting using zinc finger nucleases. *Nat. Biotechnol.* 23 967–973. 10.1038/nbt1125 16082368

[B115] Pourcel C. Salvignol G. Vergnaud G. (2005). CRISPR elements in Yersinia pestis acquire new repeats by preferential uptake of bacteriophage DNA, and provide additional tools for evolutionary studies. *Microbiology* 151 653–663. 10.1099/mic.0.27437-0 15758212

[B116] Qi L. S. Larson M. H. Gilbert L. A. Doudna J. A. Weissman J. S. Arkin A. P. (2013). Repurposing CRISPR as an RNA-guided platform for sequence-specific control of gene expression. *Cell* 5 1173–1183. 10.1016/j.cell.2013.02.022 23452860 PMC3664290

[B117] Qi W. Zhu T. Tian Z. Li C. Zhang W. Song R. (2016). High-efficiency CRISPR/Cas9 multiplex gene editing using the glycine tRNA-processing system-based strategy in maize. *BMC Biotechnol.* 16:58. 10.1186/s12896-016-0289-2 27515683 PMC4982333

[B118] Qiu Z. Kang S. He L. Zhao J. Zhang S. Hu J. (2018). The newly identified heat-stress sensitive albino 1 gene affects chloroplast development in rice. *Plant Sci.* 267 168–179. 10.1016/j.plantsci.2017.11.015 29362095

[B119] Ran F. A. Hsu P. D. Lin C. Y. Gootenberg J. S. Konermann S. Trevino A. E. (2013). Double nicking by RNA-guided CRISPR Cas9 for enhanced genome editing specificity. *Cell* 154 1380–1389. 10.1016/j.cell.2013.08.021 23992846 PMC3856256

[B120] Ren B. Yan F. Kuang Y. Li N. Zhang D. Zhou X. (2018). Improved base editor for efficiently inducing genetic variations in rice with CRISPR/Cas9-guided hyperactive hAID mutant. *Mol. Plant* 11 623–626. 10.1016/j.molp.2018.01.005 29382569

[B121] Ren C. Liu X. Zhang Z. Wang Y. Duan W. Li S. (2016). CRISPR/Cas9-mediated efficient targeted mutagenesis in Chardonnay (Vitis vinifera L.). *Sci. Rep.* 6:32289. 10.1038/srep32289 27576893 PMC5006071

[B122] Reyon D. Tsai S. Q. Khayter C. Foden J. A. Sander J. D. Joung J. K. (2012). FLASH assembly of TALENs for high-throughput genome editing. *Nat. Biotechnol.* 30 460–465. 10.1038/nbt.2170 22484455 PMC3558947

[B123] Riordan S. M. Heruth D. P. Zhang L. Q. Ye S. Q. (2015). Application of CRISPR/Cas9 for biomedical discoveries. *Cell Biosci.* 5:33. 10.1186/s13578-015-0027-9 26137216 PMC4487574

[B124] Ron M. Kajala K. Pauluzzi G. Wang D. Reynoso M. A. Zumstein K. (2014). Hairy root transformation using *Agrobacterium rhizogenes* as a tool for exploring cell type-specific gene expression and function using tomato as a model. *Plant Physiol.* 166 455–469. 10.1104/pp.114.239392 24868032 PMC4213079

[B125] Sánchez-León S. Gil-Humanes J. Ozuna C. V. Giménez M. J. Sousa C. Voytas D. F. (2018). Low-gluten, nontransgenic wheat engineered with CRISPR/Cas9. *Plant Biotechnol. J.* 16 902–910. 10.1111/pbi.12837 28921815 PMC5867031

[B126] Sanjana N. E. Shalem O. Zhang F. (2014). Improved vectors and genome-wide libraries for CRISPR screening. *Nat. Methods* 11 783–784. 10.1038/nmeth.3047 25075903 PMC4486245

[B127] Shalem O. Sanjana N. E. Hartenian E. Shi X. Scottm D. A. Mikkelsenm T. S. (2014). Genome-scale CRISPR/Cas9 knockout screening in human cells. *Science* 343 84–87. 10.1126/science.1247005 24336571 PMC4089965

[B128] Shan Q. Wang Y. Li J. Zhang Y. Chen K. Liang Z. (2013). Targeted genome modification of crop plants using a CRISPR/Cas system. *Nat. Biotechnol.* 31 686–688. 10.1038/nbt.2650 23929338

[B129] Shen L. Hua Y. Fu Y. Li J. Liu Q. Jiao X. (2017). Rapid generation of genetic diversity by multiplex CRISPR/Cas9 genome editing in rice. *Science Chin. Life Sci.* 60 506–515. 10.1007/s11427-017-9008-8 28349304

[B130] Shen L. Wang C. Fu Y. Wang J. Liu Q. Zhang X. (2018). QTL editing confers opposing yield performance in different rice varieties. *J. Int. Plant Biol.* 60 89–93. 10.1111/jipb.12501 27628577

[B131] Shi J. Gao H. Wang H. Lafitte H. R. Archibald R. L. Yang M. (2017). ARGOS8 variants generated by CRISPR-Cas9 improve maize grain yield under field drought stress conditions. *Plant Biotechnol. J.* 15 207–216. 10.1111/pbi.12603 27442592 PMC5258859

[B132] Shimatani Z. Kashojiya S. Takayama M. Terada R. Arazoe T. Ishii H. (2017). Targeted base editing in rice and tomato using a CRISPR-Cas9 cytidine deaminase fusion. *Nat. Biotechnol.* 35 441–443. 10.1038/nbt.3833 28346401

[B133] Shimatani Z. Nishizawa-Yokoi A. Endo M. Toki S. Terada R. (2015). Positive–negative-selection-mediated gene targeting in rice. *Front. Plant Sci.* 5:748. 10.3389/fpls.2014.00748 25601872 PMC4283509

[B134] Shmakov S. Abudayyeh O. O. Makarova K. S. Wolf Y. I. Gootenberg J. S. Semenova E. (2015). Discovery and functional characterization of diverse class 2 CRISPR/Cas systems. *Mol. Cell.* 60 385–397. 10.1016/j.molcel.2015.10.008 26593719 PMC4660269

[B135] Sontheimer E. J. Wolfe S. A. (2015). Cas9 gets a classmate. *Nat. Biotechnol.* 33 1240–1241. 10.1038/nbt.3426 26650011

[B136] Soyk S. Müller N. A. Park S. J. Schmalenbach I. Jiang K. Hayama R. (2017). Variation in the flowering gene SELF PRUNING 5G promotes day-neutrality and early yield in tomato. *Nat. Genet.* 49 162–168. 10.1038/ng.3733 27918538

[B137] Sternberg S. H. Redding S. Jinek M. Greene E. C. Doudna J. A. (2014). DNA interrogation by the CRISPR RNA-guided endonuclease Cas9. *Nature* 507 62–67. 10.1038/nature13011 24476820 PMC4106473

[B138] Sun Y. Zhang X. Wu C. He Y. Ma Y. Hou H. (2016). Engineering herbicide-resistant rice plants through CRISPR/Cas9-mediated homologous recombination of acetolactate synthase. *Mol. Plant* 9 628–631. 10.1016/j.molp.2016.01.001 26768120

[B139] Tambe A. East-Seletsky A. Knott G. J. Doudna J. A. O’Connell M. R. (2018). RNA binding and HEPN nuclease activation are decoupled in CRISPR-Cas13a. *bioRxiv* 190603. 10.1016/j.celrep.2018.06.105 30044970 PMC6085867

[B140] Tang X. Liu G. Zhou J. Ren Q. You Q. Tian L. (2018). A large-scale whole-genome sequencing analysis reveals highly specific genome editing by both Cas9 and Cpf1 nucleases in rice. *bioRxiv* 292086. 10.1186/s13059-018-1458-5 29973285 PMC6031188

[B141] Tang X. Zheng X. Qi Y. Zhang D. Cheng Y. Tang A. (2016). A single transcript CRISPR-Cas9 system for efficient genome editing in plants. *Mol. Plant* 9 1088–1091. 10.1016/j.molp.2016.05.001 27212389

[B142] Tian S. Jiang L. Cui X. Zhang J. Guo S. Li M. (2018). Engineering herbicide-resistant watermelon variety through CRISPR/Cas9-mediated base-editing. *Plant Cell Rep.* 37 1353–1356. 10.1007/s00299-018-2299-0 29797048

[B143] Tian S. Jiang L. Gao Q. Zhang J. Zong M. Zhang H. (2017). Efficient CRISPR/Cas9-based gene knockout in watermelon. *Plant Cell Rep.* 36 399–406. 10.1007/s00299-016-2089-5 27995308

[B144] Ueta R. Abe C. Watanabe T. Sugano S. S. Ishihara R. Ezura H. (2017). Rapid breeding of parthenocarpic tomato plants using CRISPR/Cas9. *Sci. Rep.* 7:507. 10.1038/s41598-017-00501-4 28360425 PMC5428692

[B145] Upadhyay S. K. Kumar J. Alok A. Tuli R. (2013). RNA guided genome editing for target gene mutations in wheat. *G3* 3 2233–2238. 10.1534/g3.113.008847 24122057 PMC3852385

[B146] Van Eck J. Kirk D. D. Walmsley A. M. (2006). Tomato (Lycopersicum esculentum). *Methods Mol. Biol.* 343 459–473. 16988368 10.1385/1-59745-130-4:459

[B147] Vander O. J. Jore M. M. Westra E. R. Lundgren M. Brouns S. J. (2009). CRISPR-based adaptive and heritable immunity in prokaryotes. *Trends Biochem. Sci.* 34 401–407. 10.1016/j.tibs.2009.05.002 19646880

[B148] Veres A. Gosis B. S. Ding Q. Collins R. Ragavendran A. Brand H. (2014). Low incidence of off-target mutations in individual CRISPR-Cas9 and TALEN targeted human stem cell clones detected by whole-genome sequencing. *Cell Stem Cell* 15 27–30. 10.1016/j.stem.2014.04.020 24996167 PMC4082799

[B149] Voytas D. F. (2013). Plant genome engineering with sequence-specific nucleases. *Annu. Rev. Plant Biol.* 64 327–350. 10.1146/annurev-arplant-042811-105552 23451779

[B150] Wang H. Yang H. Shivalila C. S. Dawlaty M. M. Cheng A. W. Zhang F. (2013). One-step generation of mice carrying mutations in multiple genes by CRISPR/Cas-mediated genome engineering. *Cell* 153 910–918. 10.1016/j.cell.2013.04.025 23643243 PMC3969854

[B151] Wang M. Mao Y. Lu Y. Wang Z. Tao X. Zhu J. K. (2018). Multiplex gene editing in rice with simplified CRISPR-Cpf1 and CRISPR-Cas9 systems. *J. Int. Plant Biol.* 60 626–631. 10.1111/jipb.12667 29762900

[B152] Wang W. Pan Q. He F. Akhunova A. Chao S. Trick H. (2018). Transgenerational CRISPR-Cas9 activity facilitates multiplex gene editing in allopolyploid wheat. *CRISPR J.* 1 65–74. 10.1089/crispr.2017.0010 30627700 PMC6319321

[B153] Wang X. Tu M. Wang D. Liu J. Li Y. Li Z. (2018). CRISPR/Cas9-mediated efficient targeted mutagenesis in grape in the first generation. *Plant Biotechnol. J.* 16 844–855. 10.1111/pbi.12832 28905515 PMC5866948

[B154] Wang Y. Liu X. Ren C. Zhong G. Y. Yang L. Li S. (2016). Identification of genomic sites for CRISPR/Cas9-based genome editing in the vitis vinifera genome. *BMC Plant Biol.* 16:96. 10.1186/s12870-016-0787-3 27098585 PMC4839089

[B155] Webber P. (2014). Does CRISPR/Cas open new possibilities for patents or present a moral maze? *Nat. Biotechnol.* 32 331–333. 10.1038/nbt.2843 24714479

[B156] Weeks D. P. Spalding M. H. Yang B. (2016). Use of designer nucleases for targeted gene and genome editing in plants. *Plant Biotechnol. J.* 14 483–495. 10.1111/pbi.12448 26261084 PMC11388832

[B157] Wilson L. O. W. O’Brien A. R. Bauer D. C. (2018). The current state and future of CRISPR-Cas9 gRNA design tools. *Front. Pharmacol.* 9:749. 10.3389/fphar.2018.00749 30050439 PMC6052051

[B158] Wolter F. Edelmann S. Kadri A. Scholten S. (2017). Characterization of paired Cas9 nickases induced mutations in maize mesophyll protoplasts. *Maydica* 62:11.

[B159] Woo J. W. Kim J. Kwon S. I. Corvalán C. Cho S. W. Kim H. (2015). DNA-free genome editing in plants with preassembled CRISPR/Cas9 ribonucleoproteins. *Nat. Biotechnol.* 33 1162–1164. 10.1038/nbt.3389 26479191

[B160] Xie K. Minkenberg B. Yang Y. (2015). Boosting CRISPR/Cas9 multiplex editing capability with the endogenous tRNA-processing system. *Proc. Natl. Acad. Sci. U.S.A.* 112 3570–3575. 10.1073/pnas.1420294112 25733849 PMC4371917

[B161] Xie K. Yang Y. (2013). RNA-guided genome editing in plants using a CRISPR–Cas system. *Mol. Plant* 6 1975–1983. 10.1093/mp/sst119 23956122

[B162] Xie S. Shen B. Zhang C. Huang X. Zhang Y. (2014). sgRNAcas9: a software package for designing CRISPR sgRNA and evaluating potential off-target cleavage sites. *PLoS One* 9:e100448. 10.1371/journal.pone.0100448 24956386 PMC4067335

[B163] Xing H. L. Dong L. Wang Z. P. Zhang H. Y. Han C. Y. Liu B. (2014). A CRISPR/Cas9 toolkit for multiplex genome editing in plants. *BMC Plant Biol.* 14:327. 10.1186/s12870-014-0327-y 25432517 PMC4262988

[B164] Xu R. Li H. Qin R. Wang L. Li L. Wei P. (2014). Gene targeting using the *Agrobacterium tumefaciens*-mediated CRISPR/Cas system in rice. *Rice* 7:5. 10.1186/s12284-014-0005-6 24920971 PMC4052633

[B165] Yan F. Kuang Y. Ren B. Wang J. Zhang D. Lin H. (2018). Highly efficient A⋅ T to G⋅ C base editing by Cas9n-guided tRNA adenosine deaminase in rice. *Mol. Plant* 11 631–634. 29476918 10.1016/j.molp.2018.02.008

[B166] Yu Q. H. Wang B. Li N. Tang Y. Yang S. Yang T. (2017). CRISPR/Cas9-induced targeted mutagenesis and gene replacement to generate long-shelf life tomato lines. *Sci. Rep.* 7:11874. 10.1038/s41598-017-12262-1 28928381 PMC5605656

[B167] Zetsche B. Gootenberg J. S. Abudayyeh O. O. Slaymaker I. M. Makarova K. S. Essletzbichler P. (2015). Cpf1 is a single RNA-guided endonuclease of a class 2 CRISPR-Cas system. *Cell* 163 759–771. 10.1016/j.cell.2015.09.038 26422227 PMC4638220

[B168] Zhang F. LeBlanc C. Irish V. F. Jacob Y. (2017). Rapid and efficient CRISPR/Cas9 gene editing in Citrus using the YAO promoter. *Plant Cell Rep.* 36 1883–1887. 10.1007/s00299-017-2202-4 28864834

[B169] Zhang H. Zhang J. Wei P. Zhang B. Gou F. Feng Z. (2014). The CRISPR/Cas9 system produces specific and homozygous targeted gene editing in rice in one generation. *Plant Biotechnol. J.* 12 797–807. 10.1111/pbi.12200 24854982

[B170] Zhang J. Zhang H. Botella J. R. Zhu J. K. (2018). Generation of new glutinous rice by CRISPR/Cas9-targeted mutagenesis of the Waxy gene in elite rice varieties. *J. Int. Plant Biol.* 60 369–375. 10.1111/jipb.12620 29210506 PMC5938116

[B171] Zhang Y. Zhang F. Li X. Baller J. A. Qi Y. Starker C. G. (2013). Transcription activator-like effector nucleases enable efficient plant genome engineering. *Plant Physiol.* 161 20–27. 10.1104/pp.112.205179 23124327 PMC3532252

[B172] Zhao Y. Zhang C. Liu W. Gao W. Liu C. Song G. (2016). An alternative strategy for targeted gene replacement in plants using a dual-sgRNA/Cas9 design. *Sci. Rep.* 6:23890. 10.1038/srep23890 27033976 PMC4817149

[B173] Zheng Q. Cai X. Tan M. H. Schaffert S. Arnold C. P. Gong X. (2014). Precise gene deletion and replacement using the CRISPR/Cas9 system in human cells. *Biotechniques* 57 115–124. 10.2144/000114196 25209046

[B174] Zhou H. Liu B. Weeks D. P. Spalding M. H. Yang B. (2014). Large chromosomal deletions and heritable small genetic changes induced by CRISPR/Cas9 in rice. *Nucleic Acids Res.* 42 10903–10914. 10.1093/nar/gku806 25200087 PMC4176183

[B175] Zhu J. Song N. Sun S. Yang W. Zhao H. Song W. (2016). Efficiency and inheritance of targeted mutagenesis in maize using CRISPR-Cas9. *J. Genet. Genomics* 43 25–36. 10.1016/j.jgg.2015.10.006 26842991

[B176] Zong Y. Wang Y. Li C. Zhang R. Chen K. Ran Y. (2017). Precise base editing in rice, wheat and maize with a Cas9-cytidine deaminase fusion. *Nat. Biotechnol.* 35 438–440. 10.1038/nbt.3811 28244994

